# Influence of ecological and edaphic factors on biodiversity of soil nematodes

**DOI:** 10.1016/j.sjbs.2021.02.046

**Published:** 2021-02-24

**Authors:** Rawhat Un Nisa, Aadil Yousuf Tantray, Nazia Kouser, Kaisar Ahmad Allie, Shaheen Majeed Wani, Saud A. Alamri, Mohammed Nasser Alyemeni, Leonard Wijaya, Ali Asghar Shah

**Affiliations:** a Nematode Biodiversity & Genomics Research Lab. BGSB University, Rajouri 185234, India; b Institute of Biological and Environmental Sciences, University of Aberdeen, Aberdeen AB243UU, UK; c Botany and Microbiology Department, College of Science, King Saud University, Riyadh, Saudi Arabia; d Department of Biology, Institut Teknologi Sumatera, Jalan Terusan Ryacudu, Way Hui, Jati Agung, South Lampung 35365, Indonesia

**Keywords:** Ecological factors, Indices, Nematode diversity, Soil nutrient, Soil monitoring

## Abstract

Nematodes are the most diverse and highly significant group of soil-inhabiting microorganisms that play a vital role in organic material decomposition and nutrient recycling.

Diverse geographical locations and environmental gradients show a significant impact on the diversity of nematodes. Present study aims to assess the effects of ecological (altitude, temperature, moisture) and edaphic (soil pH, nutrients, soil patches) factors on the soil nematode diversity and structure at five different landscape patches (forests, apple orchards, rice fields, pastures, and alpine zone) from ten different sites of Kashmir valley (India). Differences in the altitudinal gradients results in the shift of generic nematode population. Among the soil patches, highest nematode diversity was observed in forest soil and least in alpine soil; however, bacteriovorous nematodes dominated all the soil patches. The temperature and moisture have a significant effect on nematode diversity, the highest nematode trophic levels were observed above 21°C temperature, and 30% moisture. Nematode abundance decreased from alkaline to acidic pH of the soil. Soil nutrients such as, nitrogen (N) and phosphorus (P) have shown a detrimental effect in nematode richness at each site, where nematode diversity and richness of genera were higher at abundant soil N and P but decreased at low soil nutrients. Ecological indices like diversity index (DI), Shannon-Wiener Index (H'), enrichment index (EI), and maturity Index (MI) values demonstrated forest soil more favourable for nematodes and high soil health status than other soil patches. This study suggested that these indices may be helpful as soil monitoring tools and assessing ecosystem sustainability and biodiversity.

## Abbreviations

Shannon-Wiener Index: H′
Pielou’s Index: J′
Margalef Index: MgI
Maturity Index: MI
Nematode Channel Ratio: NCR
Nitrogen: N
Phosphorous: P
Plant Parasitic Nematode: PPN
Metre above Sea Level: m.a.s.l
Enrichment Index: EI
Channel Index: CI
Structural Index: SI
Wasilewska Index: WI

## 1 Introduction

Recently researchers have submitted the “Earth Microbiome Project” which comprises microbial compositional profiles of 27,751 samples from 97 independent studies and produced 2.2 billion DNA sequences of evolutionarily conserved genes (Thompson et al., 2017). This microbial breakthrough provides diverse insights from archaeal domains and highlights the environmental and geographic status of both terrestrial and aquatic ecosystems. Despite the invariant nematode body plan, they are showing astounding diversity. The expected nematode species extent was estimated to be more than one million, yet only about 30,000 species have been defined (Kiontke and Fitch, 2013). Due to the presence of plant and animal parasitic species, the phylum Nematoda has profound impact on agricultural products and human health. Nematodes have infected approximately half of the world's population and ~8–15% crop loss worldwide at the cost of at least $80 billion (Kiontke and Fitch, 2013). Hence, it is the need of the hour for biologists to know the nematode community structure along with varying natural ecological and edaphic factors for a sustainable life of both flora and fauna.

Diversity of living organisms including nematodes changes according to the geographical locations due to changing ecological and edaphic factors. In terrestrial soil ecosystem, nematodes constitute leading microfauna, but very little is known about the factors (ecological and edaphic) regulating their population distribution even in well-studied forest lands and agricultural fields (Hanel, 1993). Ecological factors like altitude, temperature, and precipitation are the main factors controlling terrestrial nematode diversity. Soil edaphic and climatic factors such as moisture, temperature, and pH are essential for nematode community structure (Griffiths et al., 2003). While moving along the range of altitude, plant, and animal diversity changes, but the effect is more prominent in the micro diversity of plants and animals. These changes occur due to the change in temperature and humidity along with the altitude. Bakonyi et al. (2007) found that structure and diversity of the nematode communities are more sensitive to little fluctuations in soil moisture and temperature. Temperature is an essential abiotic factor for the biology and development of nematodes as they are poikilothermic organisms (Perry, 2002). Temperature changes affect the distribution of nematodes and result in short generation time (Kamra and Sharma, 2000, McSorley, 2003, Treonis and Wall, 2005). Nematodes were found both at high and low temperatures and even at zero% relative humidity (Wall and Virginia, 1999). Some nematode species are sensitive to low temperatures (Sohlenius and Boström, 1999) and need more time for development than optimum temperature (Charchar and Santo, 2001). Soil nematodes depend on soil moisture for the safety of their soft bodies and its available resources for free-living nematodes (Liu et al., 2009). A few studies have shown that the soil moisture and soil patch have an essential role in sustaining nematode life in dry habitats (Liang and Steinberger, 2001, Joa et al., 2009, Song et al., 2016). Some reports have mentioned an impact of soil moisture, and temperature on nematode populations like *Helicotylenchus* in Guava orchard (Sen et al., 2008) and *Ditylenchus* in Groundnut (De, 1990), and a positive correlation was found with increasing soil water (Todd et al., 1999). Yeates et al. (2002) found that nematodes are greatly affected by soil moisture content. It was reported that soil dehydration must be avoided, or soil moisture should be kept at a level that would be sufficient for the least nematode activity (Radová and Trnková, 2010). Bakonyi and Nagy (2000) suggested that temperature is relatively more essential than the soil moisture content in shifting the nematode community.

Although, not only ecological factors influence nematode diversity, but edaphic characteristics also have a great impact on their structure and abundance. Nematodes occur in soil surfaces throughout the world with greater abundance in sub-arctic regions than in tropical and temperate areas, and vegetation cover indices are the best predictors of herbivore-dominated communities, while edaphic factors such as, pH and sand content are the strong interpreters of bacterivores communities (Van Den Hoogen et al., 2019). Soil type may decide the type of nematode species presence according to the water holding capacity and mineral composition of the soil. The physical and chemical characteristics of soil predominantly determine the community structure of nematodes (Goralczyk, 1998). Soil physiognomies and soil pH determine functional characteristics and population dynamics of invertebrates, which can be easily seen in certain life forms. Low pH affects nematode community organization and other components of the soil food web (Matute et al., 2013). In some related studies, pH between 5 and 7 seems favorable for nematode population growth, whether plant-parasites or free-living both in laboratory and field conditions (Warner, 2009). Meanwhile, the addition of organic manure and inorganic fertilizers changes soil features like pH, texture, porosity and results in enhanced diversity of free-living nematodes (Clark et al., 1998, Mulder et al., 2003). Intensification in bacterivorous and fungivorous nematodes and decline in plant-parasitic nematodes occur by the addition of organic matter (Bohlen and Edwards, 1994, Griffin et al., 1996). Phosphorus (P) and Nitrogen (N) content of soil greatly affects the nematode community and abundance of some nematode groups. The application of fertilizers increases the biological activity of the soil and thus enhances microbial activity along with nematode populations (Forge et al., 2003). Previous studies revealed that N concentration not only affects aboveground ecosystem processes but also belowground processes such as mineralization and microbial biomass (Fierer et al., 2012). High nematode populations and their rapid life cycle may be helpful in soil nutrient cycling.

Besides, the diversity of soil nematodes is not properly documented because of their small body size and complexity in extraction. There is no report on the realistic diversity of free-living nematodes of the Kashmir valley due to its diverse environmental conditions. It is generally believed that change in the soil microbial diversity occurs due to the ecological and edaphic factors of the soil. However, the unique region-based diversity in the ecological system necessitates more comprehensive studies encompassing diverse ecosystems in different regions to arrive at sustainable conclusion. So, the present study is aimed to investigate the impact of ecological factors like altitude, temperature, and moisture; and edaphic factors like soil pH, nutrients, and soil patches on the soil nematode diversity in diverse microclimates among the five common soil patches – forest, apple orchards, rice fields, pastures, and alpine zone. The valuable outcome of this study may be helpful to nematologists and agriculturalists to sustain ecosystems at zero environmental cost.

## 2 Materials and methods

### 2.1 Study sites and soil sampling

**Fig. 1:**
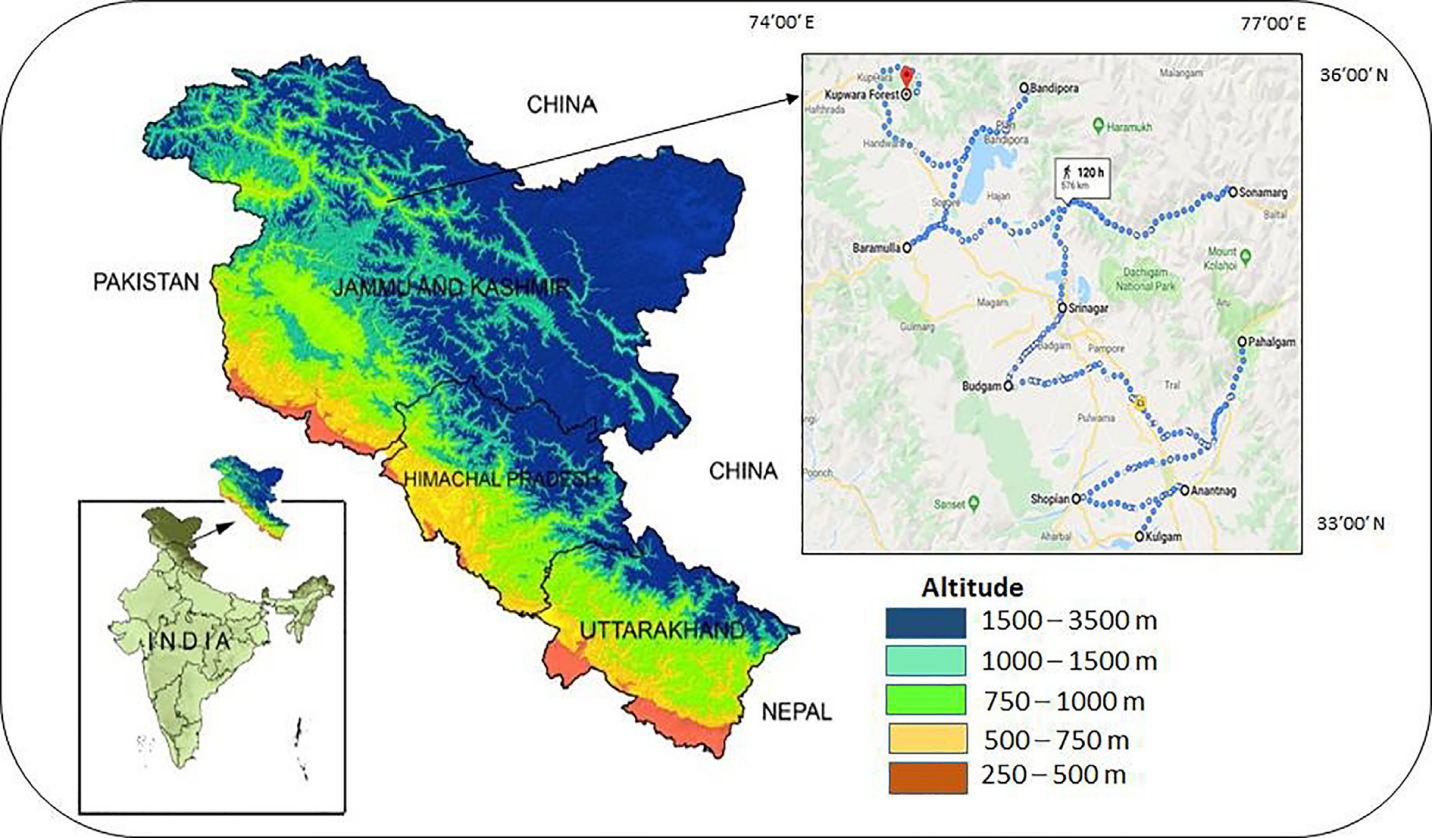
Selection of experimental sites for collection of nematode soil samples in Kashmir valley. Ten sites were represented in the figure: Kulgam, Anantnag, Shopian, Pahalgam, Budgam, Srinagar, Sonmarg, Baramulla, Bandipora and Kupwara forest from an altitude range of 500–1500 m.a.s.l.

The present study was undertaken in Kashmir valley, which lies within the geographical coordinates of 33′00′-36′00′N and 74′00′-77′00′E. Five different terrestrial landscape patches were selected (forests, apple orchards, rice fields, pastures, and alpine zone) within the altitudinal gradients of 500–1500 m.a.s.l. A total of 150 samples were collected following the method of Southy (1974). Samples were collected at an average distance of 5 km^2^ at ten different sites and each sample was taken as an independent replicate. The sampling sites of the study were selected in three altitude zones 500–750, 751–1000 and 1001–1500 m.a.s.l and three temperature ranges −1 to 10, 11–20, and 21–32°C (Fig. 1). Within each site, three independent replicates of the soil samples were collected from each landscape patch. At the time of sampling, the average temperature (°C) and relative moisture (%) of each study site were recorded with the help of a digital thermometer and moisture analyzer, respectively. For each sample, 500 mg soil was collected below 5–10 cm of ground-surface with a shovel and placed in separate airtight bags to retain the moisture. The sampling bags were labeled with necessary information like locality, date of collection, and the sample type. The collected soil samples were analyzed for nematode assay after isolation from the soil at Nematode Biodiversity and Genomics Research lab, Department of Zoology, BGSB University Rajouri, India.

#### 2.1.1 Nematode extraction and identification

The collected soil samples were thoroughly mixed with water to obtain a composite suspension. The complex soil suspension was passed through coarse sieve having mesh size of 10 mm to remove any stone, roots, or any lumps, etc. The soil was well mixed to get uniform distribution of nematodes. Sample was also then passed through fine sieve to get smaller soil particles. For the current study 50 ml volume of soil suspension was used for extraction of nematodes. Nematodes were isolated by modified Cobb sieving and decantation method and then Bearmann’s funnel technique.

For identification, microscopic glass-slides were prepared from each sample, containing enough soil nematodes. The isolated nematodes were identified up to a generic level, which was carried out under an Olympus microscope by using morphological features of the nematode genera. For each identified nematode genera, trophic groups were allocated according to Yeates et al. (1993) and c-p groups were allocated according to Bongers (1990).

#### 2.1.2 Counting of nematodes

Syracuse counting dish was used for counting the nematodes. The homogenous suspension was made by bubbling with pipette systematically before taking 2 ml of this in the Syracuse dish for counting. For each sample, counting was done almost three times, and then the mean of it was obtained. The final nematode population was attained by multiplying the final quantity of nematode suspension (50 ml) with mean nematode number and dividing by quantity of suspension used for counting that is 2 ml.

#### 2.1.3 Soil pH, moisture, nitrogen and phosphorus analysis

Before measuring the pH of the soil samples, the pH meter was calibrated and 20 g of soil from each sample was dissolved in 100 ml of distilled water. The soil suspension was stirred well for half an hour with a magnetic stirrer and then the pH reading of each sample was recorded. The moisture content was determined by the gravimetric method. Moisture content (%) = Weight of wet sample – the weight of dry sample × 100/Weight of dry sample. The nitrogen (N) and phosphorus (P) contents of soil samples were determined with the help of Energy Dispersive X-Ray Spectroscopy (EDS) from the Department of Horticulture, Soil and leaf analysis laboratory, Rajbagh Srinagar and Sarwar soil nutrient analysis Digital Laboratory Bamnoo Pulwama (PVT).

### 2.2 Diversity and community analysis

The diversity analysis of nematodes was carried by calculating; frequency (N) –Number of samples in which the genus was present; absolute frequency (AF%) –frequency of genus/total number of samples counted × 100; density (D) –number of nematodes of the genus obtained in all samples/ total number of samples collected; and relative density (RD%) –mean density of genus/sum of mean density of all the nematode genera × 100.

The community analysis of nematodes was carried by calculating nematode indices like; Shannon-Wiener Index (H′), which indicates the nematode species diversity in a community. H′= −∑ Pi ln Pi, where pi is the proportion of each taxon in the total population (Shannon, 1948). The species evenness was determined by Pielou's evenness index (J') which is closely related to species dominance. *J*′ = *H*′*/*ln *S*, where S is the taxa number (Pielou, 1966). Species richness was measured by Margalef Index (MgI) and was calculated as, *MgI = (G-1)/*ln *(n),* where *G* is the total genera number and *n* is the total number of individuals (Margalef, 1963). An environmental disturbance in soil was calculated by Maturity Index (MI). MI was calculated after allocating colonizer-persister (c–p) class values (Bongers et al., 1997). The c*–*p classes were specified based on characteristics ranging from colonizers (small life cycle with high reproduction rate & tolerate disturbances) to persisters (long life cycle with low reproduction rate & sensitive to disturbances) from 1 to 5 on a scale, meaning thereby that if a species is a strong colonizer, its c–p class would be 1 whereas strong persister has c–p class 5. MI value helps in calculating the ecological successional status of a soil community. High MI indicates persister nematode species and stable soil conditions where a low MI shows a disturbed system or highly enriched soil due to fertilizers. It was calculated as; *X  = Σvi × fi/n,* where *vi = c-p* value of the family, *fi-*frequency of family *i* in sample and *n* is the total number of individuals in a sample.

The functional structure of the community was measured by Wasilewska Index (WI), Enrichment Index (EI), Channel Index (CI), Nematode Channel Ratio (NCR), and Structural Index (SI). The WI represents the ratio of bacterial feeders (*BF*) plus fungal feeders (*FF*) to plant-parasites (*PP*) as, *WI = (BF + FF)/PP* (Wasilewska, 1994). The NCR is the ratio of the biomass of bacterivorous to fungivorous & bacterivorous nematodes. Higher values designate more fungal decomposition than bacterial decomposition i.e., *NCR = B/B + F*, where *B* ─ the abundance of bacterivorous nematodes and *F*- the abundance of fungivorous nematodes. The CI represents the fungal participation in decomposition channels of soil food webs. High value signifies dominated fungal feeding decomposition whereas, low values indicate dominated bacterial decomposition pathway. CI = 100* (Fu_2_*0.8/Ba_1_*3.2 + Fu*0.8) (Ferris et al., 2001). Enrichment Index **(**EI**)** is calculated as the biomass of opportunistic bacterivorous (*Ba1* and *Ba2*) and fungivorous (*Fu2*) nematodes that rise from the decomposition of organic matter (Ferris et al., 2001) and Structural Index (SI) indicates the state of the food web affected by disturbance or stress. High SI value indicates ecosystem stability, whereas low values represent environmental disturbances (Ferris et al., 2001). SI = 100* s/s + b where, *s = Ba_n_ + Pr_n_ + Fu_n_ + Om_n_, n = 3*–*5* and *b = Ba_2+_ Fu_2_* (Ba-bacteriovorous, Pr-Predatory, Fu-fungivorous & Om-omnivorous nematodes).

### 2.3 Statistical analysis

Analysis of variance was represented in different sets for each ecological and edaphic factor of the study, and data were generally expressed as the mean ± SD. SPSS 17.0 and SigmaPlot 12.0 Systat Softwares were used for statistical analysis. For analysis, a one-way ANOVA model was used separately for each factor, where the abundance of nematode group as response and ecological (altitude, temperature, and moisture) and edaphic (pH, N and P content) traits as factors. The significance between nematode groups was considered at *P* ≥ 0.05 (Student’s *t*-test). Principal Component Analysis (PCA) by performed by MINITAB 14.0 and correlation of nematode frequency between ecological and edaphic factors was carried by R Studio using *P* ≥ 0.05 for significance.

## 3 Results

### 3.1 Nematode diversity along with the altitude

**Fig. 2:**
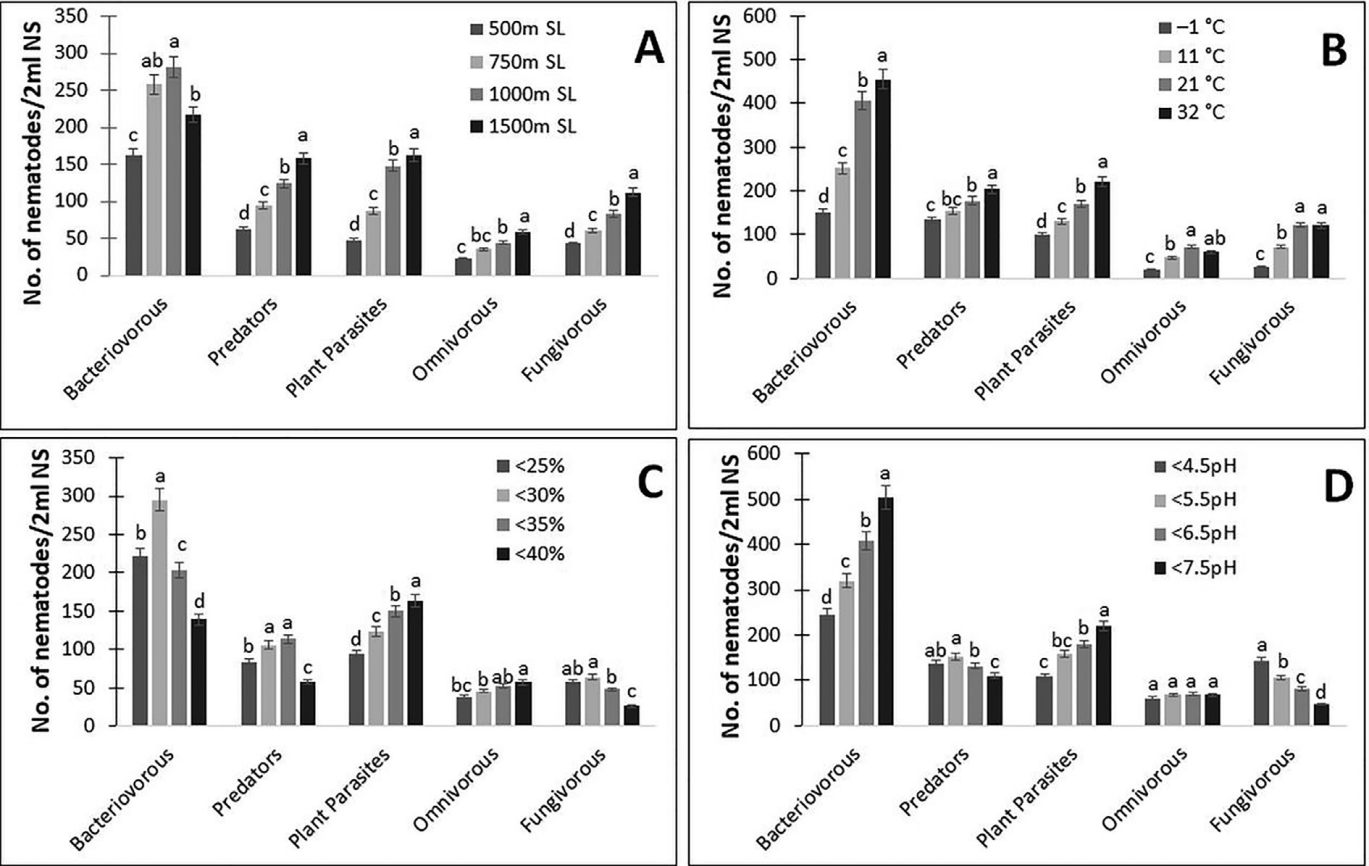
Nematode trophic groups under different ecological and edaphic factors (A) Nematodes diversity at different altitudes; (B) Nematodes diversity at different temperatures; (C) Nematodes diversity at different moistures and (D) Nematodes diversity at different soil pH are represented in figure (NS ─ Nematode Soil). Bars represent the mean of 30 soil samples (±SD) and different letters on the bars show the significance of a range of the factors at P ≥ 0.05 (Student’s *t*-test).

The diversity of nematodes changes randomly among different trophic groups along with the altitude. Bacteriovorous nematodes are found to be more abundant than other trophic groups (Fig. 2A). Nematode diversity was found to be highest at 1000–1500 m.a.s.l. as was true for bacterivorous nematodes at 1000 m.a.s.l. and as far as diversity of other nematode groups like predators, plant parasites, omnivorous and fungivorous are concerned, they are highest at 1500 m.a.s.l.

#### 3.1.1 Change in nematode diversity with temperature and moisture

Nematode diversity and abundance increased linearly with increasing temperature and moisture of terrestrial soils. The highest nematode population was found at 21–32°C and the least populations were found at temperatures below 1°C in all trophic groups. Bacteriovorous nematodes were found to exist in a wide range of temperatures and omnivorous groups were least prominent among all trophic groups at different observation sites having a narrow range of temperatures (Fig. 2B). Variable diversity of nematodes was observed concerning the moisture content in different habitats. Bacteriovorous nematodes were found most abundant in differential moisture contents, whereas the least abundance was observed for fungivorous nematodes. Different trophic groups were found with significant populations at different soil moisture content. Bacteriovorous and fungivorous nematodes were significant at <30% soil moisture, predatory nematodes at <35% and, plant parasites, and omnivores nematodes at <40% soil moisture (Fig. 2C).

#### 3.1.2 Nematode diversity along with a range of pH

Dramatic changes were observed in nematode structural populations at a wide range of pH among different trophic groups. There was a linear increase of nematode abundance in bacterivorous and plant-parasitic nematodes, but the nematode number decreased in fungivorous with an increase in pH. There was no significant change in the nematode population of omnivorous nematodes with pH change (Fig. 2D).

#### 3.1.3 Trophic nematode structure in different landscape patches

**Fig. 3:**
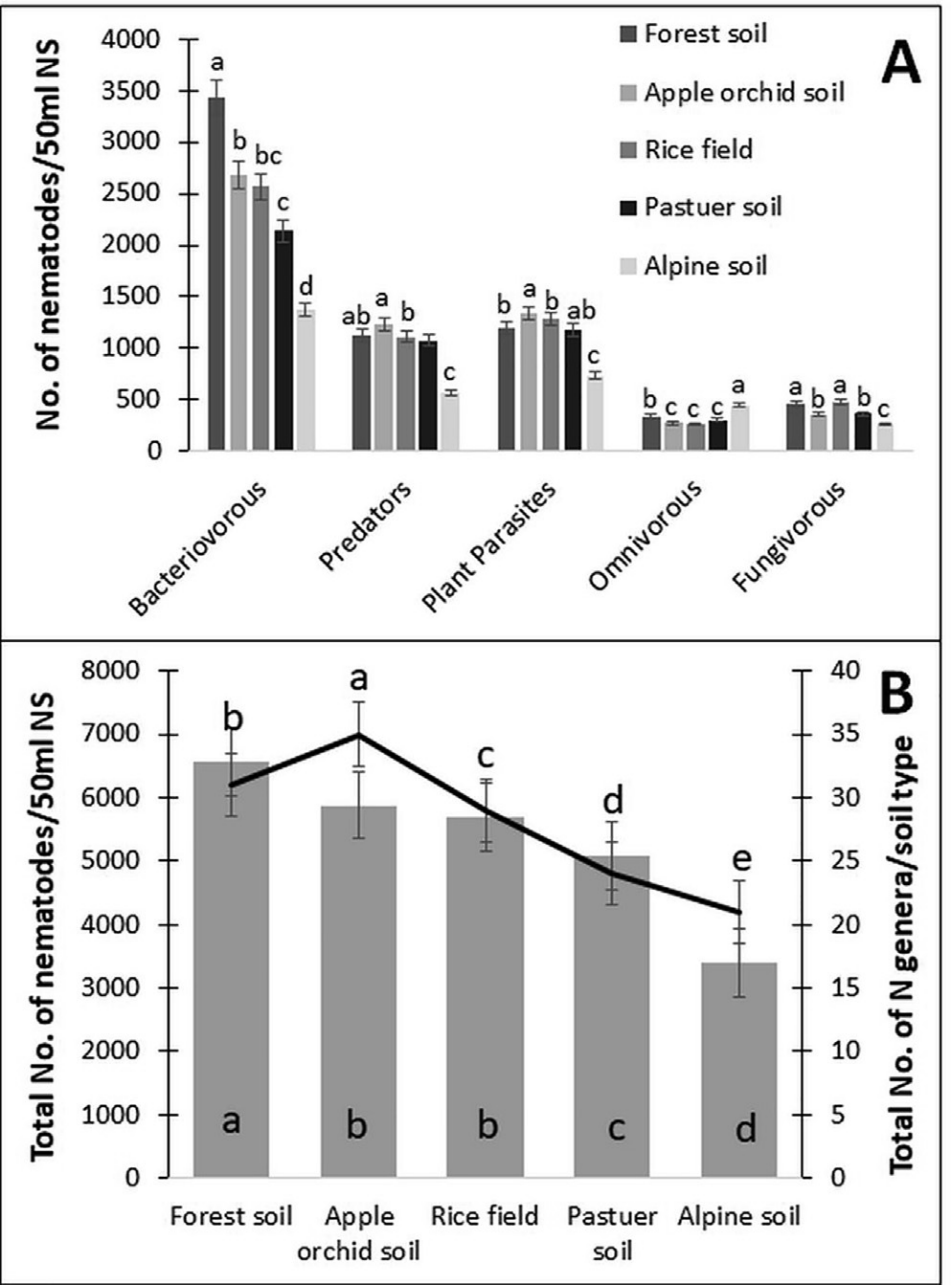
Nematode populations of different trophic groups and number of genera in five different landscape patches (A) Number of nematodes in 50 ml soil nematode solution and (B) Total number of nematodes and genera in landscape patches (NS – Nematode Soil). Bars represent the mean of 30 soil samples (±SD) and different letters on bars show the significance among landscape patches at P ≥ 0.05 (Student’s *t*-test).

The significance of the nematode population was diversified among different landscape patches. Bacteriovorous nematodes were significant in forest soil, predatory nematodes, and plant-parasitic nematodes in apple orchard soil, omnivorous in alpine soil and fungivorous nematodes were significant in rice fields (Fig. 3A). Among soil patches, highest nematode abundance was found in forest soil, and a greater number of nematode genera was reported in soil samples collected from apple orchards. The least abundance was observed in alpine soil samples and other landscape patches (Fig. 3B). Bacteriovorous covered almost 33% of nematode populations and among landscape patches; forest and apple orchard soils constitute nearly 50% of identified nematode diversity.

#### 3.1.4 Variation of nematode diversity with soil N and P

**Fig. 4:**
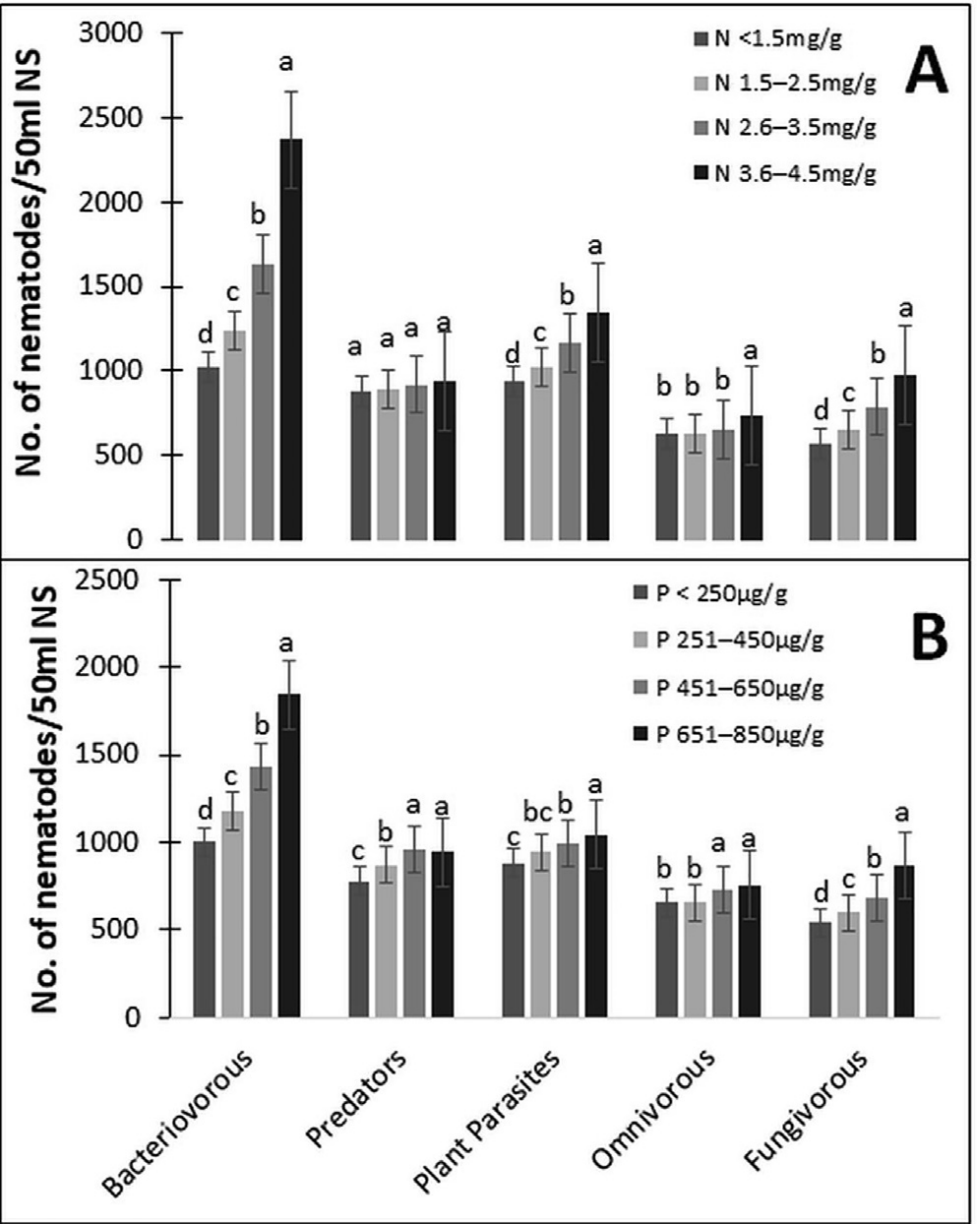
Change in populations of nematode trophic groups with changing soil nutrients. (A) Nematode populations at different soil nitrogen (N) content. (B) Nematode populations at different soil phosphorous (P) content (NS ─ Nematode Soil). Bars represent the mean of 30 soil samples (±SD) and different letters on the bars show the significance among landscape patches at P ≥ 0.05.

Nematode population structure changed with changing soil N and P content. With increasing soil N content, bacterivorous increased significantly but omnivorous have shown no change in their population with changing soil N content. Nematode population was more prominent at 3.6–4.5 mg/g soil N content but least at <1.5 mg/g soil N content (Fig. 4A). Changing soil P content depicted similar results to that of N. There was a significant increase of the nematode population in each trophic group with increasing soil P content. The highest nematode abundance was found at 651–850 µg/g soil P content and lowest in soil samples with <250 µg/g soil P content (Fig. 4B). Bacteriovorous trophic group was at the highest range, followed by plant-parasitic during both N and P observations in all soil samples.

### 3.2 Nematode abundance and density in soil samples

**Fig. 5:**
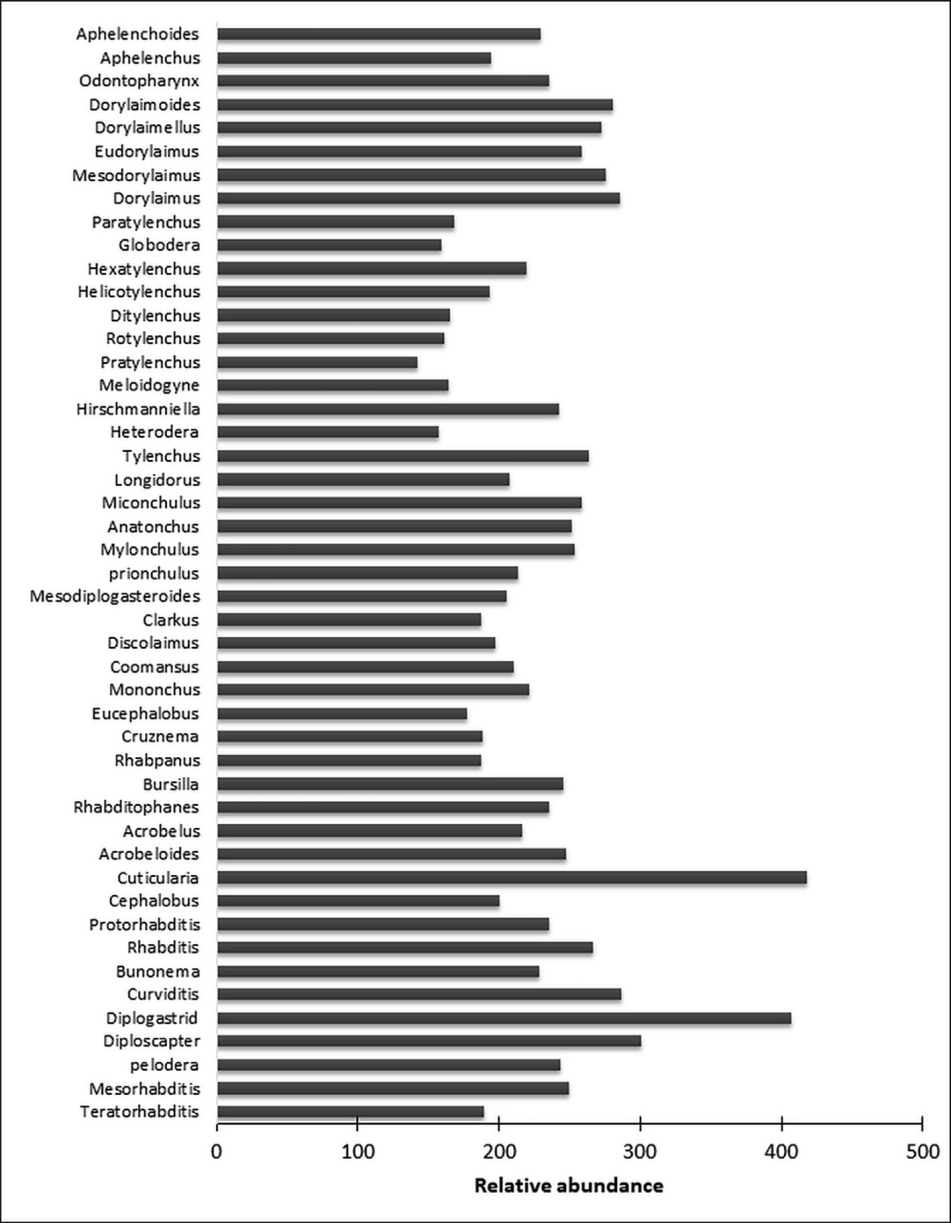
Relative abundance of nematode genera in 150 soil samples of different ecological sites.

There was diverse nematode abundance in soil samples at all sites. Forty-seven nematode genera were identified in 150 soil samples at ten different sites. Among these identified genera, *Cuticularia* was with the highest abundance followed by *Diploscapter* and *Pratylenchus* with the lowest abundance among all identified genera. *Meloidogyne*, *Longidorus*, and *Rhabditis* like genera were found with average abundance in all soil samples (Fig. 5). Density and relative density also varied among different genera in different landscape patches. The density of *Pratylenchus* was significant in forest soil, *Eucephalobus* in apple orchard soil, *Rotylenchus* in rice fields and, *Ditylenchus* in pasture and alpine soils. Other statistical parameters like frequency, absolute frequency, and relative density also showed dynamic results (Supplementary Table 1).

### 3.3 Principle component analysis on ecological factors

**Fig. 6:**
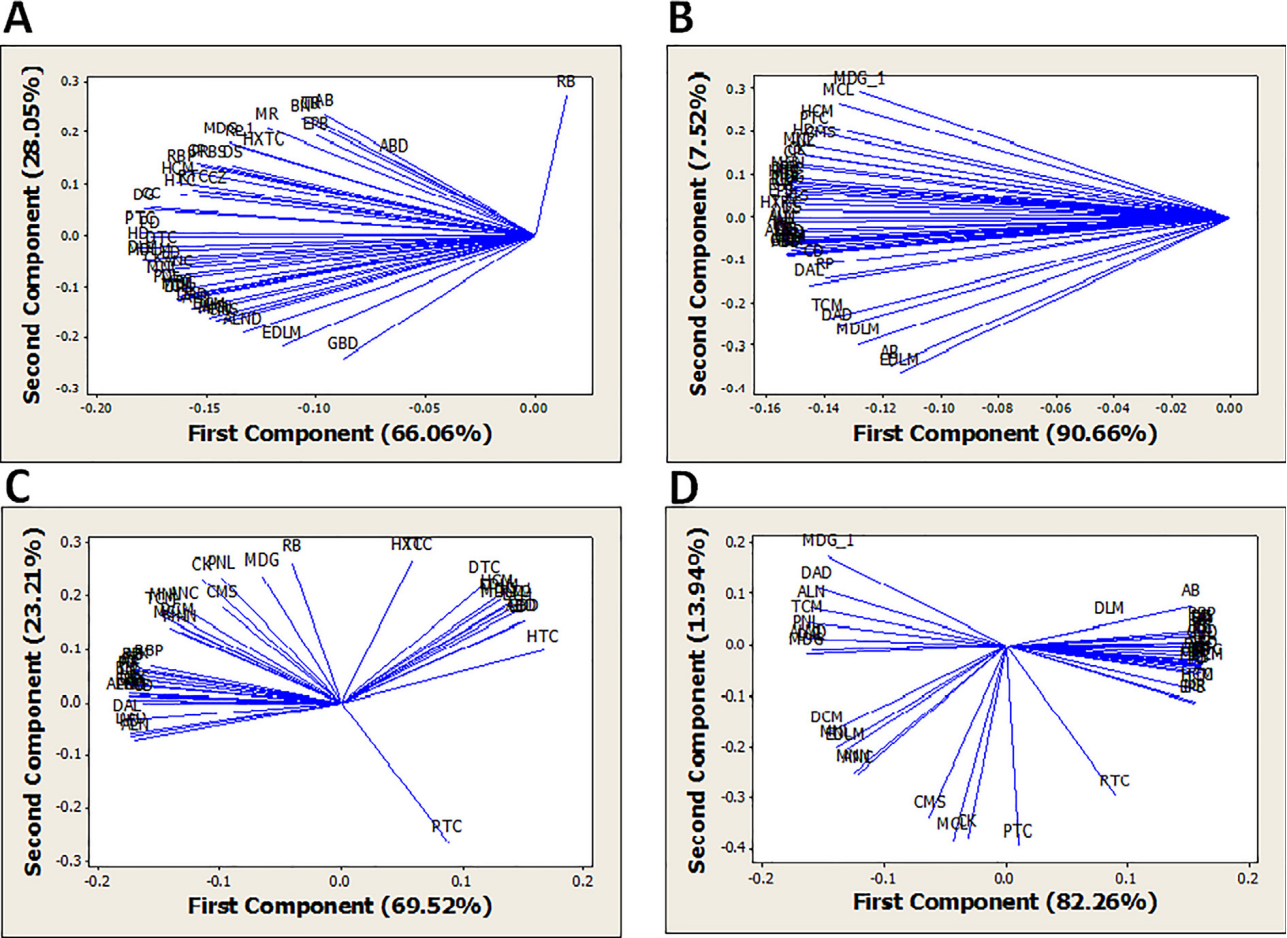
Principle Component Analysis (PCA) of forty-seven nematode genera at different ecological and edaphic factors (A) Variance at a range of altitude, (B) Variance at a range of temperature, (C) Variance at a range of soil moisture, and (D) Variance at a range of soil pH.

Variation of genera deciphered from principal component analysis (PCA) based on four ecological parameters ─ altitude, temperature, moisture, and pH of terrestrial soil samples. Genus *Rhabditis* was with maximum variation among genera at a range of altitude and moisture whereas, genus *Meloidogyne* at a range of temperature and pH (Fig. 6: Supplementary Table 2). Temperature and pH of the soil will be determined as responsible ecological factors for significant diversity variation among identified genera.

### 3.4 Diversity and community indices

The diversity and community indices of nematodes of different landscape patches represented the soil structure and stability. Shannon’s diversity index was highest in alpine soil and there were little changes in other landscape patches, but the nematode evenness was significant in forest soil (Table 1). Margalef's richness index represented rice field soil as the most significant nematode adopting soil whereas, the maturity index showed it as disturbed soil (low MI value) and alpine soil as nematode persisting soil. The nematode channel ratio and enrichment index were prominent in apple orchard soil whereas, the structural index in forest soil and channel index in pasture soil.

**Table 1:** Nematode diversity and community indices of five different landscape patches with diverse ecological and edaphic characters.

Indices	Forest Soil	Apple orchard Soil	Rice field Soil	Pasture Soil	Alpine Soil
*Shannon Weiner Index (H’)*	3.40 ± 0.12c	3.50 ± 0.14b	3.20 ± 0.10d	3.19 ± 0.09d	3.79 ± 0.16a
*Pielou’s Index (J’)*	1.00 ± 0.04a	1.00 ± 0.03ab	0.96 ± 0.03c	0.95 ± 0.02bc	0.99 ± 0.05b
*Wasilewska Index (WI)*	1.85 ± 0.08c	4.40 ± 0.15a	1.00 ± 0.05 cd	0.90 ± 0.04d	3.33 ± 0.13b
*Margalef Index (MgI)*	9.12 ± 0.26 cd	11.02 ± 0.31b	14.28 ± 0.43a	9.61 ± 0.27c	8.35 ± 0.22d
*Maturity Index (MI)*	2.90 ± 0.14b	1.90 ± 0.11 cd	1.50 ± 0.09d	2.20 ± 0.12c	3.70 ± 0.14a
*Nematode Channel Ratio (NCR)*	0.69 ± 0.07b	0.81 ± 0.09a	0.58 ± 0.06c	0.50 ± 0.06d	0.80 ± 0.09ab
*Enrichment Index (EI)*	69.23 ± 3.50bc	69.57 ± 2.79a	58.30 ± 2.34c	50.00 ± 2.55d	61.90 ± 3.31b
*Structural Index (SI)*	75.00 ± 4.22a	52.94 ± 3.58c	50.00 ± 3.42d	71.40 ± 4.13b	50.00 ± 2.97d
*Channel Index (CI)*	6.67 ± 0.21c	5.45 ± 0.18 cd	15.80 ± 0.27b	20.00 ± 0.32a	4.43 ± 0.14d

Values are mean of 30 replicates (±SD) of each index of nematodes. Within each row different letters after the mean values show the significance among landscape patches at P ≥ 0.05.

## 4 Discussion

The diversity of nematodes and the other micro-organisms depends upon the ecological and edaphic factors of their habitat. Altitude, temperature, and moisture like ecological factors play a vital role; soil pH and nutrients also greatly affect nematode populations. As expected, nematode diversity changes randomly along with the range of altitude but maximum diversity was found at 1000–1500 m.a.s.l. with maximum predatory, plant-parasitic, omnivorous, and fungivorous found at 1500 m.a.s.l. However, contradictory results were detected for various organisms of different taxonomic groups involving soil creatures like mites in which a decrease in species richness with altitude was found (Hodkinson, 2005, McCain and Grytnes, 2010, Vittoz et al., 2010, Nagy et al., 2012, Mumladze et al., 2015). As reported, nematodes occupy every ecosystem, they can colonize even harsh environments like the Antarctic or high-altitude biotopes (Yeates, 2010). Interestingly our results corroborate with those of Körner (2003) where maximum diversity was reported at the mid to high altitude that may be due to increased soil moisture at higher altitude (Fig. 2A). Bakonyi and Nagy (2000) found the leading effect of moisture on nematode richness in Hungarian grassland, even though the temperature was more significant for the soil nematode community structure. Other studies reported that free water in the soil is the regulating factor for nematode activity, and most authors reported its role in increasing nematode population (Landesman et al., 2011). Wallace (1963), found that high moisture condition inhibits nematode movement which may be responsible for decreased reproduction under such conditions. According to Wallace (1963) soil moisture, soil type, and aeration are inter-associated, and oxygen replenishment is slower in saturated soils than well-aerated soils.

According to Sylvain et al. (2014) in mesic, xeric, arid savannahs and polar deserts rise in soil moisture results in the decline of nematode communities of all trophic groups excluding grasslands and Jornada desert where no effect of moisture was found. Our observations showed the negative association of soil moisture with bacterivorous, predatory, and fungivores nematodes. Moisture and soil temperature can either affect the density of plant parasitic nematodes in a positive way (Verschoor et al., 2001) or may not affect (Smolik and Dodd, 1983). *Tylenchorhynchus* and *Xiphinema* densities are affected greater than *Pratylenchus* in terms of soil temperature and moisture (Griffin et al., 1996). The density of *Eudorylaimus* and *Aporcelaimellus* was enhanced with irrigation in citrus, so at the maximum irrigation rate omnivorous constitute 11–19% of the whole nematode community (Porazinska et al., 1998). Soil temperature has a great influence on nematode growth and affects the structure of nematode communities (Bakonyi et al., 2007). Drop in soil moisture and the rise in soil temperature results in a decrease in nematode population (Bakonyi et al., 2007). The present study shows a positive effect of temperature on the density of nematodes of all the five trophic groups. Many workers described that seasonal population variation of nematodes regarding their minimum and maximum growth due to numerous climatic conditions which are either consistent or inconsistent with the current study, yet they at last agree at one point that the soil moisture, temperature, rainfall, etc. have a significant effect on the growth of nematode population (Kumar, 2002, Sen et al., 2008). Highest population density of few endo – and ectoparasitic nematodes were reported during monsoon with a maximum temperature of 24–37°C which shows compatibility with the current study (Sabir, 2000). Maximum nematode populations were recorded during high moisture and low soil temperature (July) whereas minimum nematode populations were found throughout pre and post-monsoon seasons with a range of high to low soil temperature and moisture (Sen, 2017). The influence of temperature on the growth and development of nematodes have great significance. Munteanu (2017) reported that the best temperature range for nematodes was 15–25°C. Previous studies found that J2 of *Meloidogyne fallax* gets well hatched at low temperature (15°C) than higher temperatures (20°C and 25°C) similar to that of *M. chitwoodi* (Khan et al., 2014). Previous studies found that all the nematode groups (bacterivorous, fungivores, predatory, plant-parasitic) associated with *Brassica rapa* were negatively correlated with pH and temperature and tend to be thermophilic and acidophilic (Matute et al., 2013).

Soil chemical and ecological properties which vary in different habitats influenced nematode abundance and diversity (Spann and Schumann, 2010). Wang et al. (2009) found that increased soil acidity results in faster reproduction of *Meloidogyne* species. Both positive and negative relationship of *Meloidogyne* species and *R. solanacearum* with soil physiognomies were found signifying their part in the host-pathogen relationship (Desaeger and Rao, 2000, Wang et al., 2004). Soils with acidic pH enhance soil microbes and increase the growth and reproduction of root-knot nematodes (Kesba and Al-Shalaby, 2008). Regardless of both positive and negative correlation of soil pH with abundance, the results specify the role of soil pH in prompting nematode diversity (Zhong and Cai, 2007). Adamtey et al. (2016) reported a decrease in soil pH by regular use of fertilizers that also affects the soil microbe and nematode diversity (Zhong et al., 2010). Very few numbers of omnivorous nematodes were found at pH less than 4. At pH 2.7 maximum omnivorous were absent except a few *Eudorylaimus* were present but a reliable number of *Eudorylaimus* and *Aporcelaimellus* were found at pH > 3.7 (Háněl, 2001). At acidic pH of 3.7–4.2, *Eudorylaimus* were absent but found at sites with higher pH (Dmowska and Kozłowska, 1988). Our results show an increased density of bacterivorous and PPNs nematodes at high pH and decreased density of fungivorous at high pH but no significant consequence of pH on omnivorous nematodes. Soil pH can change soil microfauna by disturbing soil microbial events as testified by Rocha et al. (2006). Earlier studies revealed the decreased soil pH by consistent application of mineral fertilizers (Adamtey et al., 2016) disturb the soil microfauna and diversity of nematode population (Zhong et al., 2010). Diverse environments with different ecological and environmental factors results in different frequency and density of nematodes (Khatoon et al., 2001).

**Fig. 7:**
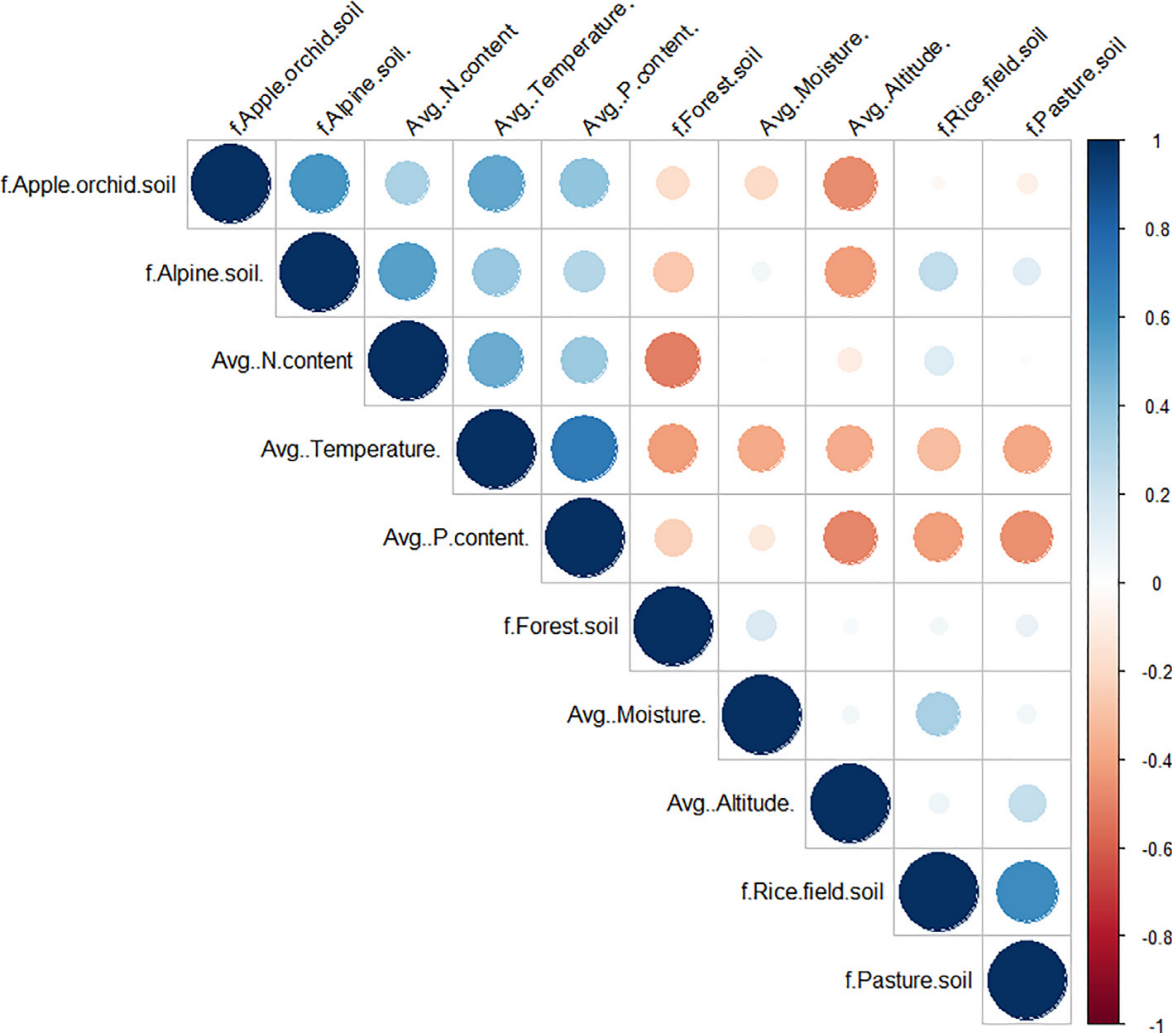
Correlation of ecological and edaphic factors with the frequency of nematodes in different landscape patches. Blue-colored spots show a positive correlation whereas; orange-colored spots show a negative correlation.

The N and P contents of the soil have a great role in regulating the diversity of nematodes and other micro-fauna of the soil. A strong positive correlation between fungivores and PPNs with N and P signifies that rise in any of these macronutrients will increase these nematode groups. Our results coincide with earlier studies which found the increased N results in great populations of PPNs (de Melo Santana-Gomes et al., 2013). Hatchability of *Meloidogyne exigua* was enhanced by adding P as Potassium phosphate and results in an increased population of *M. exigua* in soil with high potassium or rock phosphate was found (Salgado et al., 2007). Current study signifies that N was strongly correlated with nematode density, but greater N concentration does not always signify greater nematode density. In farmland ecosystems beyond a certain level of excess N results in a decrease of nematode diversity (XU et al., 2007). The reason may be that high N concentration results in increased growth of microbes, causing condensed nematode growth that leads to competition between nematodes and results in a decrease of nematode diversity (Post et al., 1985). The present study shows that physicochemical and edaphic factors of soils were highly correlated and showed a significant impact on the community structure of soil nematodes, signifying that nematodes have finely divided niches concerning the physicochemical and edaphic factors that change with altitude. Our studies found that temperature, nitrogen, phosphorus, and pH were all vital environmental factors influencing the nematode community structure (Fig. 7). In the alpine zone, soil nematodes showed a strong positive correlation with average N, T, and P content while a negative correlation with altitude. Rice field nematodes are negatively correlated with average T and P content, but positively correlated with average moisture. In pasture soil, nematodes showed a strong negative correlation with average T and P while a positive correlation with average altitude. No significant correlation was shown by forest soil nematodes.

Agricultural practices like tillage, crop alternation, and irrigation alter microbial and nematode trophic assembly and ultimately affects the soil ecosystem (Yeates and Bongers, 1999). Excellent nematode diversity is found in grasslands (Todd et al., 2006) and can offer ecosystem services such as pest regulation, inhibition of nutrient losses, and greenhouse gas discharges related to agricultural systems (Culman et al., 2010, Glover et al., 2010). Our results are comparable with these findings and showed the lowest diversity in grasslands and highest in the alpine region. Earlier studies reported that bacterivores nematodes having c-p value1 are more opportunistic in response than other nematodes to resource enrichment (Ferris et al., 2004). External application of organic material like fertilizers and crop residues results in increased abundance of bacterivores nematodes (c-p value 1) in particular (Bulluck Iii et al., 2002, Ferris et al., 2004). The EI represents the richness of enrichment opportunists comparative to the richness of basal taxa, measures the degree of resource enrichment, and acts as an indicator of soil productivity (Ferris et al., 2001). Our results were similar to findings of Ferris et al. (2001) which reported a lower value of EI in grasslands and higher in agricultural systems (Table 1). For nematode ecological indices, EI was enhanced by N but did not affect SI. High EI found in apple orchards and forests show a dominant bacterial decomposition pathway (Ugarte et al., 2013). EI value represents enhanced obtainability of resources of the soil ecosystem and the response of primary decomposers to the resources, which seemed directly associated to the collective amounts of N mineralized in the soil (Ferris and Matute, 2003). The present study reported the decrease in CI values by N addition (Table 1), which is similar to earlier reports of Azpilicueta et al., 2014, Pan et al., 2015, which showed decreased CI value in N added plots than control plots. For decomposition pathways, CI is a good indicator (Ferris et al., 2001). Lower CI denotes that N addition pushes the soil food web to bacterial decomposition pathways. MI value indicates the soil disruption developed by the application of fertilizers (Bongers et al., 1997). In present study lowest MI value in rice fields and highest in the alpine region has been observed, which may be due to the addition of fertilizers (N) in rice fields resulting in the dominance of bacterivorous nematodes. The decline in MI suggests the declining structure of the nematode community as the soil food web complexity decreases with enhancing N deposition.

In the present study, 47 nematode genera were documented from 10 different sites. Bacteriovores constitute the highest genera number with *Cuticularia* having the highest abundance and *Pratylenchus* the lowest (Fig. 5). *Rhabditida* showed dominance both in terms of genera and abundance, followed by plant-parasitic and predatory nematodes. *Mononchids* and *Dorylaimids* were found to be more sensitive than other nematode groups to disturbances and to the physico chemical conditions of the soil environment as was also reported by (Forge and Simard, 2001). These results are also in agreement with earlier reports that *Dorylaimid* populations in the nematode communities are sensitive to disturbances like agricultural practices, such as ploughing, pesticides, and fertilizers, and are, therefore, used as indicators of environmental disturbances (Sohlenius and Wasilewska, 1984). Among different landscape patches, bacterial feeders dominated in abundance and density in apple orchard soil and alpine soil, followed by plant-parasitic nematodes in rice field soil. An abundance of predatory nematodes was found because of lesser anthropogenic disturbances in forest ecosystems. However, earlier studies reported bacterivorous nematodes to be the most dominant group followed by plant parasites (Neher et al., 2005).

Diversity and community indices of nematodes are helpful to analyze the efficiency and stability of the soil, feeding pathways, life strategy to colonize new habitat, and resistance. Shannon’s diversity index (H’) is used to describe species diversity in a community that is more reliable indicator of the evenness and abundance of the species present (Roth et al., 1994) and disturbance of the habitat. In our investigation, the H' values were highest in alpine soil (3.79) which indicate less disturbance and high evenness of this soil compared to more disturbed rice fields hence with less H' values (3.20). Pielou’s evenness index was highest in forest and apple orchard soils and represents greater evenness in these soils because of high energy production and resource distribution (Pielou, 1966). The Margalef index explains the richness of species diversity in a community (Clifford and Stephenson, 1975), which was higher in apple orchard soil and least in alpine soil. The number of nematode species may be higher in the apple orchard soil, because of a high quantity of N and P elements, and high moisture content. The mineralization dominant pathway was indicated by the Wasilewska (1994) index. The highest WI values of apple orchard soil and lowest WI values of pasture soil were surprising results of this study. As the lower values of WI proposes that the dominant pathway goes through grazing by plant-feeding nematodes and higher values suggest detrital pathway predomination. The disturbance of habitat can be measured by using the maturity index (MI) of the nematode populations (Bongers, 1990). In our experiment, the highest MI was found in alpine soil (3.70) and lowest in rice field soil (1.50), which indicates that rice field soils are more disturbed due to the applications of fertilizers and pesticides. The pH and chemical composition of soil changes regularly in rice field soils, whereas alpine soil remains undisturbed without any human interference.

Structural index (SI), Enrichment index (EI), and Channel index (CI) act as indicators for population regulation and feeding pathways response. The EI gives food web responses to existing resources, SI measures the tropical layers and potential for regulation of opportunists, and CI represents the predominant decomposition pathways (Ferris et al., 2001). In the present study, EI was highest in apple orchard soil (69.59), showing a good response of available resources and with more likely broad food-web but pasture soils fall in response, which may be due to less availability of resources. The forest soil has shown higher SI values and indicates large tropical layers and a high potential for regulation of opportunists, which was least in rice field soils. This may be because of the addition of fertilizers in the agricultural field and less disturbance of forest habitat. The CI in our study was higher in pasture soil (20.00) and the lowest in alpine soil (4.43); signifying that the food-web structure was bacterial-dominant in pasture soil followed by forest soil and rice field soil.

## 5 Conclusion

Current study showed that nematode abundance and diversity falls when moving from low to high altitude. While at low temperature, there was lower nematode abundance and diversity, but on moving from low moisture to high moisture content, the nematode population and diversity increased. In terms of soil chemistry, soil pH, N, and P content were detrimental factors for nematode populations in all the landscape patches. The nematode abundance and diversity increased while moving from acidic pH to alkaline pH and in abundant N and P contents of the soils. Forest and apple orchard soils were highly diversified with nematodes, whereas alpine soil was with less number. In all the landscape patches, bacterivorous nematodes were dominating in each ecological condition. Diversity and community indices suggested that forest soils have great efficiency in adopting nematodes and have high community structure and food-web connections whereas, rice field soil was highly disturbed and low efficiency.

## Author contributions

RUN, AAS and AYT conceived and designed the experiments; RUN and NK performed the experiments and sample test; RUN, KAA, AAS and SMW identified nematodes; SAA, MNA and LW analyzed the data and helped in revision. All the authors read and approved the final manuscript.

## Declaration of Competing Interest

The authors declare that they have no known competing financial interests or personal relationships that could have appeared to influence the work reported in this paper.
