# Supplementary material for: Influence of ecological and edaphic factors on biodiversity of soil nematodes
Source: Saudi J Biol Sci. 2021 Feb 24;28(5):3049–59. doi: 10.1016/j.sjbs.2021.02.046 (PMC8117023; doi:10.1016/j.sjbs.2021.02.046)
Supplement: Supplementary data 1 [file mmc1.docx]

**Saudi Journal of Biological Sciences**

**Title of the Manuscript: Influence of ecological and edaphic factors on biodiversity of soil nematodes**

**By**

Rawhat Un Nisa^1^, Aadil Yousuf Tantray^2^, Nazia Kouser^1^, Kaisar Ahmad Allie^1^, Shaheen Majeed Wani^1^, Ali Asghar Shah^1^ *

^1^Nematode Biodiversity & Genomics Research Lab. BGSB University, Rajouri, 185234, India

^2^Institute of Biological and Environmental Sciences, University of Aberdeen, Aberdeen, AB243UU, UK

*Corresponding author

Email: [headzoology@bgsbu.ac.in](mailto:headzoology@bgsbu.ac.in)

**Supplementary data 1:** Mean values of statistical analysis of nematode genera observed in different soil types of Kashmir

**Forest soil**

Absolute

Mean

Relative

colonizer-

No. of

Genera FrequencyFrequency Density

|  | | | | | | (c-p value) | s |
| --- | --- | --- | --- | --- | --- | --- | --- |
| *Teratorhabditis* | 10.00 | 33.33 | 80.00 | 2.67 | 62.94 | 1.00 | 12.00 |
| *Mesorhabditis* | 10.00 | 33.33 | 100.00 | 3.33 | 78.68 | 1.00 | 20.00 |
| *Pelodera* | 13.00 | 43.33 | 72.00 | 2.40 | 56.65 | 1.00 | 14.00 |
| *Diploscapter* | 14.00 | 46.67 | 120.00 | 4.00 | 94.41 | 1.00 | 22.00 |
| *Diplogastrid* | 0.00 | 0.00 | 0.00 | 0.00 | 0.00 | 0.00 | 0.00 |
| *Curviditis* | 6.00 | 20.00 | 60.00 | 2.00 | 47.21 | 1.00 | 16.00 |
| *Bunonema* | 10.00 | 33.33 | 70.00 | 2.33 | 55.07 | 1.00 | 15.00 |
| *Rhabditis* | 6.00 | 20.00 | 40.00 | 1.33 | 31.47 | 1.00 | 17.00 |
| *Protorhabditis* | 0.00 | 0.00 | 0.00 | 0.00 | 0.00 | 0.00 | 0.00 |
| *Cephalobus* | 0.00 | 0.00 | 0.00 | 0.00 | 0.00 | 0.00 | 0.00 |
| *Cuticularia* | 0.00 | 0.00 | 0.00 | 0.00 | 0.00 | 0.00 | 0.00 |
| *Acrobeloides* | 10.00 | 33.33 | 98.00 | 3.27 | 77.10 | 2.00 | 18.00 |
| *Acrobelus* | 0.00 | 0.00 | 0.00 | 0.00 | 0.00 | 0.00 | 0.00 |
| *Rhabditophanes* | 0.00 | 0.00 | 0.00 | 0.00 | 0.00 | 0.00 | 0.00 |
| *Bursilla* | 0.00 | 0.00 | 0.00 | 0.00 | 0.00 | 0.00 | 0.00 |
| *Rhabpanus* | 0.00 | 0.00 | 0.00 | 0.00 | 0.00 | 0.00 | 0.00 |
| *Cruznema* | 10.00 | 33.33 | 59.00 | 1.97 | 46.42 | 1.00 | 13.00 |
| *Eucephalobus* | 11.00 | 36.67 | 90.00 | 3.00 | 70.81 | 2.00 | 11.00 |
|  |  | 0.00 | 0.00 | 0.00 | 0.00 | 0.00 | 0.00 |
| *Mononchus* | 15.00 | 50.00 | 120.00 | 4.00 | 94.41 | 1.00 | 13.00 |
| *Coomansus* | 0.00 | 0.00 | 0.00 | 0.00 | 0.00 | 0.00 | 0.00 |
| *Discolaimus* | 16.00 | 53.33 | 140.00 | 4.67 | 110.15 | 5.00 | 6.00 |
| *Clarkus* | 14.00 | 46.67 | 135.00 | 4.50 | 106.22 | 4.00 | 7.00 |
| *Mesodiplogasteroides* | 13.00 | 43.33 | 125.00 | 4.17 | 98.35 | 1.00 | 3.00 |
| *Prionchulus* | 17.00 | 56.67 | 155.00 | 5.17 | 121.95 | 4.00 | 8.00 |
| *Mylonchulus* | 18.00 | 60.00 | 160.00 | 5.33 | 125.89 | 4.00 | 9.00 |
| *Anatonchus* | 13.00 | 43.33 | 135.00 | 4.50 | 106.22 | 4.00 | 4.00 |
| *Miconchulus* | 16.00 | 53.33 | 145.00 | 4.83 | 114.08 | 4.00 | 10.00 |
| *Longidorus* | 14.00 | 46.67 | 125.00 | 4.17 | 98.35 | 5.00 | 20.00 |
| *Tylenchus* | 15.00 | 50.00 | 135.00 | 4.50 | 106.22 | 2.00 | 22.00 |
| *Heterodera* | 14.00 | 46.67 | 120.00 | 4.00 | 94.41 | 3.00 | 13.00 |
| *Hirschmanniella* | 0.00 | 0.00 | 0.00 | 0.00 | 0.00 | 0.00 | 0.00 |
| *Meloidogyne* | 15.00 | 50.00 | 130.00 | 4.33 | 102.28 | 3.00 | 18.00 |

density

density

persister

nematodes

| *Pratylenchus* | 15.00 | 50.00 | 200.00 | 6.67 | 157.36 | 3.00 | 24.00 |
| --- | --- | --- | --- | --- | --- | --- | --- |
| *Rotylenchus* | 16.00 | 53.33 | 170.00 | 5.67 | 133.75 | 3.00 | 21.00 |
| *Ditylenchus* | 17.00 | 56.67 | 180.00 | 6.00 | 141.62 | 2.00 | 22.00 |
| *Helicotylenchus* | 0.00 | 0.00 | 0.00 | 0.00 | 0.00 | 3.00 | 0.00 |
| *Hexatylenchus* | 0.00 | 0.00 | 0.00 | 0.00 | 0.00 | 0.00 | 0.00 |
| *Globodera* | 0.00 | 0.00 | 0.00 | 0.00 | 0.00 | 0.00 | 0.00 |
| *Paratylenchus* | 17.00 | 56.67 | 143.00 | 4.77 | 112.51 | 2.00 | 17.00 |
| *Dorylaimus* | 12.00 | 40.00 | 135.00 | 4.50 | 106.22 | 4.00 | 10.00 |
| *Mesodorylaimus* | 13.00 | 43.33 | 140.00 | 4.67 | 110.15 | 4.00 | 11.00 |
| *Eudorylaimus* | 11.00 | 36.67 | 110.00 | 3.67 | 86.55 | 4.00 | 14.00 |
|  |  | 0.00 | 0.00 | 0.00 | 0.00 | 0.00 | 0.00 |
| *Dorylaimellus* | 7.00 | 23.33 | 73.00 | 2.43 | 57.44 | 4.00 | 13.00 |
| *Dorylaimoides* | 10.00 | 33.33 | 100.00 | 3.33 | 78.68 | 4.00 | 12.00 |
| *Tylencholaimus* | 0.00 | 0.00 | 0.00 | 0.00 | 0.00 | 0.00 | 0.00 |
| *Aphelenchus* | 8.00 | 26.67 | 85.00 | 2.83 | 66.88 | 2.00 | 16.00 |
| *Aphelenchoides* | 6.00 | 20.00 | 65.00 | 2.17 | 51.14 | 2.00 | 12.00 |
| **Apple orchid soil** |  |  |  |  |  |  |  |

Genera Frequency Absolute Density

Frequency

Mean density

|  | | | | | | (c-p value) |  |
| --- | --- | --- | --- | --- | --- | --- | --- |
| *Teratorhabditis* | 13.00 | 43.33 | 95.00 | 3.17 | 79.86 | 3.00 | 16.00 |
| *Mesorhabditis* | 12.00 | 40.00 | 110.00 | 3.67 | 92.47 | 1.00 | 25.00 |
| *Pelodera* | 11.00 | 36.67 | 80.00 | 2.67 | 67.25 | 1.00 | 16.00 |
| *Diploscapter* | 14.00 | 46.67 | 125.00 | 4.17 | 105.08 | 1.00 | 30.00 |
| *Diplogastrid* | 16.00 | 53.33 | 155.00 | 5.17 | 130.30 | 1.00 | 28.00 |
| *Curviditis* | 10.00 | 33.33 | 70.00 | 2.33 | 58.84 | 1.00 | 24.00 |
| *Bunonema* | 11.00 | 36.67 | 82.00 | 2.73 | 68.93 | 1.00 | 19.00 |
| *Rhabditis* | 10.00 | 33.33 | 73.00 | 2.43 | 61.37 | 1.00 | 20.00 |
| *Protorhabditis* | 14.00 | 46.67 | 140.00 | 4.67 | 117.69 | 1.00 | 25.00 |
| *Cephalobus* | 16.00 | 53.33 | 150.00 | 5.00 | 126.09 | 2.00 | 30.00 |
| *Cuticularia* | 13.00 | 43.33 | 120.00 | 4.00 | 100.87 | 2.00 | 25.00 |
| *Acrobeloides* | 11.00 | 36.67 | 99.00 | 3.30 | 83.22 | 2.00 | 22.00 |
| *Acrobelus* | 12.00 | 40.00 | 85.00 | 2.83 | 71.45 | 2.00 | 18.00 |
| *Rhabditophanes* | 11.00 | 36.67 | 90.00 | 3.00 | 75.66 | 1.00 | 20.00 |
| *Bursilla* | 11.00 | 36.67 | 98.00 | 3.27 | 82.38 | 1.00 | 35.00 |
| *Rhabpanus* | 12.00 | 40.00 | 110.00 | 3.67 | 92.47 | 1.00 | 14.00 |
| *Cruznema* | 16.00 | 53.33 | 143.00 | 4.77 | 120.21 | 1.00 | 17.00 |
| *Eucephalobus* | 17.00 | 56.67 | 160.00 | 5.33 | 134.50 | 2.00 | 16.00 |
|  | 0.00 | 0.00 | 0.00 | 0.00 | 0.00 | 0.00 | 0.00 |
| *Mononchus* | 6.00 | 20.00 | 70.00 | 2.33 | 58.84 | 1.00 | 19.00 |
| *Coomansus* | 3.00 | 10.00 | 50.00 | 1.67 | 42.03 | 4.00 | 20.00 |
| *Discolaimus* | 4.00 | 13.33 | 60.00 | 2.00 | 50.44 | 5.00 | 14.00 |
| *Clarkus* | 2.00 | 6.67 | 25.00 | 0.83 | 21.02 | 4.00 | 15.00 |
| *Mesodiplogasteroides* | 0.00 | 0.00 | 0.00 | 0.00 | 0.00 | 0.00 | 0.00 |
| *Prionchulus* | 0.00 | 0.00 | 0.00 | 0.00 | 0.00 | 0.00 | 0.00 |
| *Mylonchulus* | 8.00 | 26.67 | 70.00 | 2.33 | 58.84 | 4.00 | 16.00 |
| *Anatonchus* | 7.00 | 23.33 | 80.00 | 2.67 | 67.25 | 4.00 | 18.00 |
| *Miconchulus* | 0.00 | 0.00 | 0.00 | 0.00 | 0.00 | 0.00 | 0.00 |

Relative density

colonizer- persister

No. of nematodes

|  | 0.00 | 0.00 | 0.00 | 0.00 | 0.00 | 0.00 | 0.00 |
| --- | --- | --- | --- | --- | --- | --- | --- |
| *Longidorus* | 11.00 | 36.67 | 100.00 | 3.33 | 84.06 | 5.00 | 20.00 |
| *Tylenchus* | 0.00 | 0.00 | 0.00 | 0.00 | 0.00 | 0.00 | 0.00 |
| *Heterodera* | 0.00 | 0.00 | 0.00 | 0.00 | 0.00 | 0.00 | 0.00 |
| *Hirschmanniella* | 0.00 | 0.00 | 0.00 | 0.00 | 0.00 | 0.00 | 0.00 |
| *Meloidogyne* | 0.00 | 0.00 | 0.00 | 0.00 | 0.00 | 0.00 | 0.00 |
| *Pratylenchus* | 14.00 | 46.67 | 135.00 | 4.50 | 113.48 | 3.00 | 22.00 |
| *Rotylenchus* | 13.00 | 43.33 | 145.00 | 4.83 | 121.89 | 3.00 | 28.00 |
| *Ditylenchus* | 15.00 | 50.00 | 110.00 | 3.67 | 92.47 | 2.00 | 30.00 |
| *Helicotylenchus* | 15.00 | 50.00 | 78.00 | 2.60 | 65.57 | 3.00 | 31.00 |
| *Hexatylenchus* | 0.00 | 0.00 | 0.00 | 0.00 | 0.00 | 0.00 | 0.00 |
| *Globodera* | 0.00 | 0.00 | 0.00 | 0.00 | 0.00 | 0.00 | 0.00 |
| *Paratylenchus* | 14.00 | 46.67 | 70.00 | 2.33 | 58.84 | 2.00 | 18.00 |
|  | 0.00 | 0.00 | 0.00 | 0.00 | 0.00 | 0.00 | 0.00 |
| *Dorylaimus* | 0.00 | 0.00 | 0.00 | 0.00 | 0.00 | 0.00 | 0.00 |
| *Mesodorylaimus* | 11.00 | 36.67 | 104.00 | 3.47 | 87.42 | 4.00 | 15.00 |
| *Eudorylaimus* | 10.00 | 33.33 | 100.00 | 3.33 | 84.06 | 4.00 | 16.00 |
|  | 0.00 | 0.00 | 0.00 | 0.00 | 0.00 | 0.00 | 0.00 |
| *Dorylaimellus* | 8.00 | 26.67 | 89.00 | 2.97 | 74.82 | 4.00 | 19.00 |
| *Dorylaimoides* | 0.00 | 0.00 | 0.00 | 0.00 | 0.00 | 0.00 | 0.00 |
| *Tylencholaimus* | 9.00 | 30.00 | 100.00 | 3.33 | 84.06 | 1.00 | 16.00 |
| *Aphelenchus* | 10.00 | 33.33 | 102.00 | 3.40 | 85.74 | 2.00 | 19.00 |
| *Aphelenchoides* | 9.00 | 30.00 | 96.00 | 3.20 | 80.70 | 2.00 | 17.00 |
| **Rice field soil** |  |  |  |  |  |  |  |

Genera Frequency Absolute Density

Frequency

|  | | | | | | (c-p value) | s |
| --- | --- | --- | --- | --- | --- | --- | --- |
| *Teratorhabditis* | 4.00 | 13.33 | 40.00 | 1.33 | 0.26 | 3.00 | 9.00 |
| *Mesorhabditis* | 7.00 | 23.33 | 60.00 | 2.00 | 0.38 | 1.00 | 12.00 |
| *Pelodera* | 4.00 | 13.33 | 44.00 | 1.47 | 0.28 | 1.00 | 9.00 |
| *Diploscapter* | 8.00 | 26.67 | 80.00 | 2.67 | 0.51 | 1.00 | 12.00 |
| *Diplogastrid* | 11.00 | 36.67 | 100.00 | 3.33 | 0.64 | 1.00 | 11.00 |
| *Curviditis* | 3.00 | 10.00 | 38.00 | 1.27 | 0.24 | 1.00 | 10.00 |
| *Bunonema* | 4.00 | 13.33 | 49.00 | 1.63 | 0.31 | 1.00 | 12.00 |
| *Rhabditis* | 15.00 | 50.00 | 100.00 | 3.33 | 0.64 | 1.00 | 9.00 |
| *Protorhabditis* | 10.00 | 33.33 | 90.00 | 3.00 | 0.57 | 1.00 | 13.00 |
| *Cephalobus* | 14.00 | 46.67 | 125.00 | 4.17 | 0.80 | 2.00 | 14.00 |
| *Cuticularia* | 10.00 | 33.33 | 77.00 | 2.57 | 0.49 | 2.00 | 9.00 |
| *Acrobeloides* | 7.00 | 23.33 | 53.00 | 1.77 | 0.34 | 2.00 | 6.00 |
| *Acrobelus* | 8.00 | 26.67 | 70.00 | 2.33 | 0.45 | 2.00 | 7.00 |
| *Rhabditophanes* | 3.00 | 10.00 | 40.00 | 1.33 | 0.26 | 1.00 | 8.00 |
| *Bursilla* | 3.00 | 10.00 | 34.00 | 1.13 | 0.22 | 1.00 | 8.00 |
| *Rhabpanus* | 4.00 | 13.33 | 50.00 | 1.67 | 0.32 | 1.00 | 4.00 |
| *Cruznema* | 1.00 | 3.33 | 40.00 | 1.33 | 0.26 | 1.00 | 10.00 |
| *Eucephalobus* | 9.00 | 30.00 | 70.00 | 2.33 | 0.45 | 2.00 | 8.00 |
|  | 0.00 | 0.00 | 0.00 | 0.00 | 0.00 | 0.00 | 0.00 |
| *Mononchus* | 7.00 | 23.33 | 90.00 | 3.00 | 0.57 | 1.00 | 7.00 |
| *Coomansus* | 0.00 | 0.00 | 0.00 | 0.00 | 0.00 | 4.00 | 10.00 |
| *Discolaimus* | 1.00 | 3.33 | 20.00 | 0.67 | 0.13 | 5.00 | 4.00 |

Mean density

Relative density

colonizer- persister

No. of nematodes

| *Clarkus* | 0.00 | 0.00 | 0.00 | 0.00 | 0.00 | 4.00 | 6.00 |
| --- | --- | --- | --- | --- | --- | --- | --- |
| *Mesodiplogasteroides* | 0.00 | 0.00 | 0.00 | 0.00 | 0.00 | 1.00 | 3.00 |
| *Prionchulus* | 6.00 | 20.00 | 75.00 | 2.50 | 0.48 | 4.00 | 1.00 |
| *Mylonchulus* | 6.00 | 20.00 | 70.00 | 2.33 | 0.45 | 4.00 | 1.00 |
| *Anatonchus* | 4.00 | 13.33 | 45.00 | 1.50 | 0.29 | 4.00 | 7.00 |
| *Miconchulus* | 8.00 | 26.67 | 80.00 | 2.67 | 0.51 | 4.00 | 2.00 |
|  | 0.00 | 0.00 | 0.00 | 0.00 | 0.00 | 0.00 | 0.00 |
| *Longidorus* | 17.00 | 56.67 | 160.00 | 5.33 | 1.02 | 5.00 | 6.00 |
| *Tylenchus* | 20.00 | 66.67 | 180.00 | 6.00 | 1.15 | 2.00 | 4.00 |
| *Heterodera* | 19.00 | 63.33 | 175.00 | 5.83 | 1.12 | 3.00 | 11.00 |
| *Hirschmanniella* | 22.00 | 73.33 | 202.00 | 6.73 | 1.29 | 3.00 | 12.00 |
| *Meloidogyne* | 21.00 | 70.00 | 196.00 | 6.53 | 1.25 | 3.00 | 13.00 |
| *Pratylenchus* | 24.00 | 80.00 | 220.00 | 7.33 | 1.40 | 3.00 | 10.00 |
| *Rotylenchus* | 20.00 | 66.67 | 222.00 | 7.40 | 1.42 | 3.00 | 11.00 |
| *Ditylenchus* | 20.00 | 66.67 | 198.00 | 6.60 | 1.26 | 2.00 | 10.00 |
| *Helicotylenchus* | 16.00 | 53.33 | 170.00 | 5.67 | 1.08 | 3.00 | 8.00 |
| *Hexatylenchus* | 13.00 | 43.33 | 135.00 | 4.50 | 0.86 | 2.00 | 4.00 |
| *Globodera* | 14.00 | 46.67 | 140.00 | 4.67 | 0.89 | 3.00 | 9.00 |
| *Paratylenchus* | 18.00 | 60.00 | 160.00 | 5.33 | 1.02 | 2.00 | 9.00 |
|  | 0.00 | 0.00 | 0.00 | 0.00 | 0.00 | 0.00 | 0.00 |
| *Dorylaimus* | 12.00 | 40.00 | 0.00 | 0.00 | 0.00 | 4.00 | 8.00 |
| *Mesodorylaimus* | 10.00 | 33.33 | 130.00 | 4.33 | 0.83 | 4.00 | 7.00 |
| *Eudorylaimus* | 11.00 | 36.67 | 100.00 | 3.33 | 0.64 | 4.00 | 10.00 |
|  | 0.00 | 0.00 | 0.00 | 0.00 | 0.00 | 0.00 | 0.00 |
| *Dorylaimellus* | 14.00 | 46.67 | 135.00 | 4.50 | 0.86 | 4.00 | 6.00 |
| *Dorylaimoides* | 16.00 | 53.33 | 145.00 | 4.83 | 0.92 | 4.00 | 5.00 |
| *Tylencholaimus* | 14.00 | 46.67 | 140.00 | 4.67 | 0.89 | 1.00 | 2.00 |
| *Aphelenchus* | 16.00 | 53.33 | 153.00 | 5.10 | 0.98 | 2.00 | 1.00 |
| *Aphelenchoides* | 24.00 | 80.00 | 204.00 | 6.80 | 1.30 | 2.00 | 4.00 |
| **Pasture land** |  |  |  |  |  |  |  |

Genera Frequency Absolute Density

Frequency

|  | | | | | | (c-p value) |  |
| --- | --- | --- | --- | --- | --- | --- | --- |
| *Teratorhabditis* | 10.00 | 33.33 | 70.00 | 2.33 | 37.63 | 3.00 | 10.00 |
| *Mesorhabditis* | 9.00 | 30.00 | 94.00 | 3.13 | 50.53 | 1.00 | 14.00 |
| *Pelodera* | 8.00 | 26.67 | 60.00 | 2.00 | 32.25 | 1.00 | 10.00 |
| *Diploscapter* | 11.00 | 36.67 | 100.00 | 3.33 | 53.76 | 1.00 | 15.00 |
| *Diplogastrid* | 12.00 | 40.00 | 110.00 | 3.67 | 59.13 | 1.00 | 16.00 |
| *Curviditis* | 8.00 | 26.67 | 52.00 | 1.73 | 27.95 | 1.00 | 11.00 |
| *Bunonema* | 9.00 | 30.00 | 80.00 | 2.67 | 43.01 | 1.00 | 14.00 |
| *Rhabditis* | 11.00 | 36.67 | 82.00 | 2.73 | 44.08 | 1.00 | 13.00 |
| *Protorhabditis* | 12.00 | 40.00 | 110.00 | 3.67 | 59.13 | 1.00 | 15.00 |
| *Cephalobus* | 13.00 | 43.33 | 120.00 | 4.00 | 64.51 | 2.00 | 17.00 |
| *Cuticularia* | 6.00 | 20.00 | 85.00 | 2.83 | 45.69 | 2.00 | 11.00 |
| *Acrobeloides* | 7.00 | 23.33 | 70.00 | 2.33 | 37.63 | 2.00 | 12.00 |
| *Acrobelus* | 5.00 | 16.67 | 68.00 | 2.27 | 36.56 | 2.00 | 10.00 |
| *Rhabditophanes* | 1.00 | 3.33 | 45.00 | 1.50 | 24.19 | 1.00 | 9.00 |
| *Bursilla* | 6.00 | 20.00 | 50.00 | 1.67 | 26.88 | 1.00 | 12.00 |
| *Rhabpanus* | 7.00 | 23.33 | 58.00 | 1.93 | 31.18 | 1.00 | 10.00 |

Mean density

Relative density

colonizer- persister

No. of nematodes

| *Cruznema* | 9.00 | 30.00 | 45.00 | 1.50 | 24.19 | 1.00 | 13.00 |
| --- | --- | --- | --- | --- | --- | --- | --- |
| *Eucephalobus* | 6.00 | 20.00 | 60.00 | 2.00 | 32.25 | 2.00 | 11.00 |
|  | 0.00 | 0.00 | 0.00 | 0.00 | 0.00 | 0.00 | 0.00 |
| *Mononchus* | 12.00 | 40.00 | 110.00 | 3.67 | 59.13 | 1.00 | 9.00 |
| *Coomansus* | 9.00 | 30.00 | 100.00 | 3.33 | 53.76 | 4.00 | 12.00 |
| *Discolaimus* | 8.00 | 26.67 | 90.00 | 3.00 | 48.38 | 5.00 | 8.00 |
| *Clarkus* | 10.00 | 33.33 | 100.00 | 3.33 | 53.76 | 4.00 | 7.00 |
| *Mesodiplogasteroides* | 9.00 | 30.00 | 95.00 | 3.17 | 51.07 | 1.00 | 5.00 |
| *Prionchulus* | 8.00 | 26.67 | 80.00 | 2.67 | 43.01 | 4.00 | 2.00 |
| *Mylonchulus* | 10.00 | 33.33 | 100.00 | 3.33 | 53.76 | 4.00 | 1.00 |
| *Anatonchus* | 7.00 | 23.33 | 83.00 | 2.77 | 44.62 | 4.00 | 8.00 |
| *Miconchulus* | 16.00 | 53.33 | 140.00 | 4.67 | 75.26 | 4.00 | 2.00 |
|  | 0.00 | 0.00 | 0.00 | 0.00 | 0.00 | 0.00 | 0.00 |
| *Longidorus* | 10.00 | 33.33 | 160.00 | 5.33 | 86.01 | 5.00 | 10.00 |
| *Tylenchus* | 19.00 | 63.33 | 180.00 | 6.00 | 96.76 | 2.00 | 18.00 |
| *Heterodera* | 13.00 | 43.33 | 175.00 | 5.83 | 94.08 | 3.00 | 17.00 |
| *Hirschmanniella* | 10.00 | 33.33 | 202.00 | 6.73 | 108.59 | 3.00 | 11.00 |
| *Meloidogyne* | 11.00 | 36.67 | 196.00 | 6.53 | 105.37 | 3.00 | 18.00 |
| *Pratylenchus* | 14.00 | 46.67 | 135.00 | 4.50 | 72.57 | 3.00 | 18.00 |
| *Rotylenchus* | 12.00 | 40.00 | 160.00 | 5.33 | 86.01 | 3.00 | 20.00 |
| *Ditylenchus* | 14.00 | 46.67 | 240.00 | 8.00 | 129.02 | 2.00 | 16.00 |
| *Helicotylenchus* | 26.00 | 86.67 | 153.00 | 5.10 | 82.25 | 3.00 | 15.00 |
| *Hexatylenchus* | 10.00 | 33.33 | 120.00 | 4.00 | 64.51 | 2.00 | 18.00 |
| *Globodera* | 12.00 | 40.00 | 135.00 | 4.50 | 72.57 | 3.00 | 4.00 |
| *Paratylenchus* | 15.00 | 50.00 | 130.00 | 4.33 | 69.88 | 2.00 | 5.00 |
|  | 0.00 | 0.00 | 0.00 | 0.00 | 0.00 | 0.00 | 0.00 |
| *Dorylaimus* | 11.00 | 36.67 | 95.00 | 3.17 | 51.07 | 4.00 | 10.00 |
| *Mesodorylaimus* | 10.00 | 33.33 | 115.00 | 3.83 | 61.82 | 4.00 | 9.00 |
| *Eudorylaimus* | 7.00 | 23.33 | 98.00 | 3.27 | 52.68 | 4.00 | 15.00 |
|  | 0.00 | 0.00 | 0.00 | 0.00 | 0.00 | 0.00 | 0.00 |
| *Dorylaimellus* | 14.00 | 46.67 | 76.00 | 24.26 | 40.86 | 4.00 | 10.00 |
| *Dorylaimoides* | 16.00 | 53.33 | 84.00 | 2.80 | 45.16 | 4.00 | 11.00 |
| *Tylencholaimus* | 14.00 | 46.67 | 80.00 | 2.67 | 43.01 | 1.00 | 8.00 |
| *Aphelenchus* | 16.00 | 53.33 | 73.00 | 2.43 | 39.24 | 2.00 | 6.00 |
| *Aphelenchoides* | 24.00 | 80.00 | 65.00 | 2.17 | 34.94 | 2.00 | 5.00 |
| **Alpine Soil** |  |  |  |  |  |  |  |

Genera Frequency Absolute Density

Frequency

Mean density

|  | | | | | | (c-p value) |  |
| --- | --- | --- | --- | --- | --- | --- | --- |
| *Teratorhabditis* | 11.00 | 36.67 | 88.00 | 2.93 | 62.63 | 3.00 | 14.00 |
| *Mesorhabditis* | 12.00 | 40.00 | 95.00 | 3.17 | 67.62 | 1.00 | 20.00 |
| *Pelodera* | 10.00 | 33.33 | 76.00 | 2.53 | 54.09 | 1.00 | 14.00 |
| *Diploscapter* | 12.00 | 40.00 | 100.00 | 3.33 | 71.17 | 1.00 | 22.00 |
| *Diplogastrid* | 14.00 | 46.67 | 142.00 | 4.73 | 101.07 | 1.00 | 20.00 |
| *Curviditis* | 9.00 | 30.00 | 65.00 | 2.17 | 46.26 | 1.00 | 21.00 |
| *Bunonema* | 8.00 | 26.67 | 80.00 | 2.67 | 56.94 | 1.00 | 15.00 |
| *Rhabditis* | 7.00 | 23.33 | 80.00 | 2.67 | 56.94 | 1.00 | 18.00 |
| *Protorhabditis* | 12.00 | 40.00 | 135.00 | 4.50 | 96.09 | 1.00 | 22.00 |
| *Cephalobus* | 14.00 | 46.67 | 84.00 | 2.80 | 59.79 | 2.00 | 21.00 |

Relative density

colonizer- persister

No. of nematodes

| *Cuticularia* | 11.00 | 36.67 | 110.00 | 3.67 | 78.29 | 2.00 | 20.00 |
| --- | --- | --- | --- | --- | --- | --- | --- |
| *Acrobeloides* | 12.00 | 40.00 | 98.00 | 3.27 | 69.75 | 2.00 | 15.00 |
| *Acrobelus* | 10.00 | 33.33 | 80.00 | 2.67 | 56.94 | 2.00 | 16.00 |
| *Rhabditophanes* | 9.00 | 30.00 | 66.00 | 2.20 | 46.98 | 1.00 | 14.00 |
| *Bursilla* | 12.00 | 40.00 | 85.00 | 2.83 | 60.50 | 1.00 | 25.00 |
| *Rhabpanus* | 14.00 | 46.67 | 123.00 | 4.10 | 87.54 | 1.00 | 10.00 |
| *Cruznema* | 17.00 | 56.67 | 150.00 | 5.00 | 106.76 | 1.00 | 14.00 |
| *Eucephalobus* | 10.00 | 33.33 | 100.00 | 3.33 | 71.17 | 2.00 | 15.00 |
|  | 0.00 | 0.00 | 0.00 | 0.00 | 0.00 | 0.00 | 0.00 |
| *Mononchus* | 4.00 | 13.33 | 60.00 | 2.00 | 42.70 | 1.00 | 18.00 |
| *Coomansus* | 0.00 | 0.00 | 0.00 | 0.00 | 0.00 | 4.00 | 0.00 |
| *Discolaimus* | 3.00 | 10.00 | 35.00 | 1.17 | 24.91 | 5.00 | 10.00 |
| *Clarkus* | 6.00 | 20.00 | 55.00 | 1.83 | 39.15 | 4.00 | 12.00 |
| *Mesodiplogasteroides* | 8.00 | 26.67 | 80.00 | 2.67 | 56.94 | 1.00 | 10.00 |
| *Prionchulus* | 3.00 | 10.00 | 40.00 | 1.33 | 28.47 | 4.00 | 8.00 |
| *Mylonchulus* | 4.00 | 13.33 | 45.00 | 1.50 | 32.03 | 4.00 | 11.00 |
| *Anatonchus* | 5.00 | 16.67 | 60.00 | 2.00 | 42.70 | 4.00 | 16.00 |
| *Miconchulus* | 2.00 | 6.67 | 20.00 | 0.67 | 14.23 | 4.00 | 9.00 |
|  | 0.00 | 0.00 | 0.00 | 0.00 | 0.00 | 0.00 | 0.00 |
| *Longidorus* | 8.00 | 26.67 | 109.00 | 3.63 | 77.58 | 5.00 | 15.00 |
| *Tylenchus* | 10.00 | 33.33 | 110.00 | 3.67 | 78.29 | 2.00 | 22.00 |
| *Heterodera* | 11.00 | 36.67 | 80.00 | 2.67 | 56.94 | 3.00 | 27.00 |
| *Hirschmanniella* | 12.00 | 40.00 | 75.00 | 2.50 | 53.38 | 3.00 | 13.00 |
| *Meloidogyne* | 9.00 | 30.00 | 110.00 | 3.67 | 78.29 | 3.00 | 24.00 |
| *Pratylenchus* | 13.00 | 43.33 | 145.00 | 4.83 | 103.20 | 3.00 | 20.00 |
| *Rotylenchus* | 14.00 | 46.67 | 150.00 | 5.00 | 106.76 | 3.00 | 25.00 |
| *Ditylenchus* | 13.00 | 43.33 | 210.00 | 7.00 | 149.47 | 2.00 | 26.00 |
| *Helicotylenchus* | 21.00 | 70.00 | 140.00 | 4.67 | 99.64 | 3.00 | 29.00 |
| *Hexatylenchus* | 9.00 | 30.00 | 110.00 | 3.67 | 78.29 | 2.00 | 25.00 |
| *Globodera* | 10.00 | 33.33 | 109.00 | 3.63 | 77.58 | 3.00 | 14.00 |
| *Paratylenchus* | 13.00 | 43.33 | 100.00 | 3.33 | 71.17 | 2.00 | 17.00 |
|  | 0.00 | 0.00 | 0.00 | 0.00 | 0.00 | 0.00 | 0.00 |
| *Dorylaimus* | 7.00 | 23.33 | 75.00 | 2.50 | 53.38 | 4.00 | 15.00 |
| *Mesodorylaimus* | 9.00 | 30.00 | 93.00 | 3.10 | 66.19 | 4.00 | 14.00 |
| *Eudorylaimus* | 8.00 | 26.67 | 86.00 | 2.87 | 61.21 | 4.00 | 13.00 |
|  | 0.00 | 0.00 | 0.00 | 0.00 | 0.00 | 0.00 | 0.00 |
| *Dorylaimellus* | 8.00 | 26.67 | 83.00 | 2.77 | 59.07 | 4.00 | 16.00 |
| *Dorylaimoides* | 6.00 | 20.00 | 79.00 | 2.63 | 56.23 | 4.00 | 14.00 |
| *Tylencholaimus* | 7.00 | 23.33 | 80.00 | 2.67 | 56.94 | 1.00 | 15.00 |
| *Aphelenchus* | 7.00 | 23.33 | 78.00 | 2.60 | 55.52 | 2.00 | 17.00 |
| *Aphelenchoides* | 4.00 | 13.33 | 43.00 | 1.43 | 30.60 | 2.00 | 17.00 |
