# Supplementary material for: Influence of ecological and edaphic factors on biodiversity of soil nematodes
Source: Saudi J Biol Sci. 2021 Feb 24;28(5):3049–59. doi: 10.1016/j.sjbs.2021.02.046 (PMC8117023; doi:10.1016/j.sjbs.2021.02.046)
Supplement: Supplementary data 2 [file mmc2.docx]

**Saudi Journal of Biological Sciences**

**Title of the Manuscript: Influence of ecological and edaphic factors on biodiversity of soil nematodes**

**By**

Rawhat Un Nisa^1^, Aadil Yousuf Tantray^2^, Nazia Kouser^1^, Kaisar Ahmad Allie^1^, Shaheen Majeed Wani^1^, Ali Asghar Shah^1^ *

^1^Nematode Biodiversity & Genomics Research Lab. BGSB University, Rajouri, 185234, India

^2^Institute of Biological and Environmental Sciences, University of Aberdeen, Aberdeen, AB243UU, UK

*Corresponding author

Email: headzoology@bgsbu.ac.in

**Supplementary data 2:** Mean data of nematode genera at different ecological conditions used for principal component analysis (PCA)

|  | | pH |  | | |
| --- | --- | --- | --- | --- | --- |
| Genera | Abbreviations | < 4.5 | 4.6─5.5 | 5.6─6.5 | 6.6─7.5 |
| *Teratorhabditis* | TR | 7 | 10 | 16 | 17 |
| *Mesorhabditis* | MR | 10 | 15 | 17 | 20 |
| *Pelodera* | PD | 13 | 17 | 18 | 23 |
| *Diploscapter* | DS | 18 | 21 | 30 | 33 |
| *Diplogastrid* | DG | 32 | 35 | 44 | 50 |
| *Curviditis* | CD | 21 | 24 | 28 | 33 |
| *Bunonema* | BN | 16 | 19 | 22 | 25 |
| *Rhabditis* | RB | 9 | 13 | 17 | 20 |
| *Protorhabditis* | PR | 8 | 11 | 15 | 23 |
| *Cephalobus* | CP | 5 | 12 | 16 | 25 |
| *Cuticularia* | CC | 15 | 28 | 40 | 55 |
| *Acrobeloides* | ABD | 11 | 16 | 22 | 28 |
| *Acrobelus* | AB | 12 | 13 | 15 | 23 |
| *Rhabditophanes* | RBP | 23 | 25 | 30 | 35 |
| *Bursilla* | BS | 21 | 25 | 30 | 33 |
| *Rhabpanus* | RP | 11 | 14 | 18 | 25 |
| *Cruznema* | CZ | 8 | 10 | 14 | 17 |
| *Eucephalobus* | EPB | 6 | 12 | 16 | 18 |
| *Mononchus* | MNN | 12 | 14 | 12 | 9 |
| *Coomansus* | CMS | 9 | 12 | 11 | 8 |
| *Discolaimus* | DCM | 15 | 16 | 14 | 11 |
| *Clarkus* | CK | 7 | 10 | 9 | 7 |
| *Mesodiplogasteroides* | MDG | 16 | 15 | 13 | 11 |
| *Prionchulus* | PNL | 20 | 18 | 16 | 13 |
| *Mylonchulus* | MNL | 19 | 20 | 18 | 17 |
| *Anatonchus* | ANC | 20 | 24 | 18 | 16 |
| *Miconchulus* | MCL | 18 | 23 | 20 | 18 |
| *Longidorus* | LD | 9 | 13 | 17 | 23 |
| *Tylenchus* | TC | 13 | 17 | 23 | 30 |
| *Heterodera* | HD | 7 | 10 | 17 | 23 |
| *Hirschmanniella* | HCM | 14 | 19 | 21 | 25 |

| *Meloidogyne* | MDG | 12 | 10 | 10 | 9 |
| --- | --- | --- | --- | --- | --- |
| *Paratylenchus* | PTC | 5 | 20 | 13 | 10 |
| *Rotylenchus* | RTC | 7 | 15 | 11 | 14 |
| *Ditylenchus* | DTC | 5 | 9 | 11 | 17 |
| *Helicotylenchus* | HTC | 10 | 14 | 17 | 19 |
| *Hexatylenchus* | HXTC | 13 | 15 | 17 | 19 |
| *Globodera* | GBD | 5 | 7 | 10 | 13 |
| *Dorylaimus* | DLM | 24 | 23 | 30 | 26 |
| *Mesodorylaimus* | MDLM | 18 | 22 | 25 | 29 |
| *Eudorylaimus* | EDLM | 19 | 23 | 15 | 13 |
| *Dorylaimellus* | DAL | 35 | 30 | 27 | 15 |
| *Dorylaimoides* | DAD | 47 | 28 | 15 | 9 |
| *Tylencholaimus* | TCM | 30 | 22 | 16 | 7 |
| *Aphelenchus* | ALN | 15 | 12 | 11 | 8 |
| *Aphelenchoides* | ALND | 16 | 14 | 12 | 8 |
| *Genera* |  | Temperature  _1C | 11C | 21C | 32C |
| *Teratorhabditis* | TR | 6 | 10 | 15 | 18 |
| *Mesorhabditis* | MR | 8 | 15 | 25 | 28 |
| *Pelodera* | PD | 10 | 13 | 22 | 29 |
| *Diploscapter* | DS | 5 | 10 | 30 | 35 |
| *Diplogastrid* | DG | 12 | 20 | 35 | 38 |
| *Curviditis* | CD | 6 | 11 | 38 | 35 |
| *Bunonema* | BN | 4 | 9 | 25 | 26 |
| *Rhabditis* | RB | 13 | 20 | 25 | 27 |
| *Protorhabditis* | PR | 9 | 14 | 18 | 20 |
| *Cephalobus* | CP | 7 | 11 | 15 | 22 |
| *Cuticularia* | CC | 20 | 29 | 45 | 48 |
| *Acrobeloides* | ABD | 7 | 15 | 22 | 24 |
| *Acrobelus* | AB | 6 | 15 | 20 | 15 |
| *Rhabditophanes* | RBP | 5 | 7 | 11 | 14 |
| *Bursilla* | BS | 7 | 13 | 14 | 18 |
| *Rhabpanus* | RP | 2 | 9 | 10 | 11 |
| *Cruznema* | CZ | 14 | 18 | 19 | 25 |
| *Eucephalobus* | EPB | 9 | 13 | 18 | 22 |
| *Mononchus* | MNN | 21 | 22 | 24 | 26 |
| *Coomansus* | CMS | 23 | 25 | 25 | 28 |
| *Discolaimus* | DCM | 7 | 12 | 20 | 21 |
| *Clarkus* | CK | 14 | 16 | 17 | 20 |
| *Mesodiplogasteroides* | MDG | 13 | 15 | 16 | 18 |
| *Prionchulus* | PNL | 9 | 11 | 14 | 17 |
| *Mylonchulus* | MNL | 16 | 17 | 19 | 22 |
| *Anatonchus* | ANC | 14 | 18 | 22 | 24 |
| *Miconchulus* | MCL | 17 | 18 | 20 | 28 |
| *Longidorus* | LD | 9 | 12 | 14 | 17 |
| *Tylenchus* | TC | 13 | 18 | 20 | 25 |
| *Heterodera* | HD | 7 | 9 | 12 | 18 |
| *Hirschmanniella* | HCM | 14 | 15 | 17 | 22 |
| *Meloidogyne* | MDG | 7 | 6 | 9 | 16 |
| *Paratylenchus* | PTC | 7 | 10 | 12 | 20 |

| *Rotylenchus* | RTC | 5 | 8 | 14 | 18 |
| --- | --- | --- | --- | --- | --- |
| *Ditylenchus* | DTC | 10 | 14 | 17 | 22 |
| *Helicotylenchus* | HTC | 13 | 15 | 18 | 21 |
| *Hexatylenchus* | HXTC | 2 | 6 | 11 | 14 |
| *Globodera* | GBD | 8 | 12 | 16 | 17 |
| *Dorylaimus* | DLM | 9 | 14 | 25 | 26 |
| *Mesodorylaimus* | MDLM | 4 | 16 | 23 | 18 |
| *Eudorylaimus* | EDLM | 7 | 17 | 24 | 17 |
| *Dorylaimellus* | DAL | 9 | 14 | 25 | 23 |
| *Dorylaimoides* | DAD | 7 | 18 | 26 | 22 |
| *Tylencholaimus* | TCM | 3 | 12 | 22 | 18 |
| *Aphelenchus* | ALN | 3 | 10 | 20 | 24 |
| *Aphelenchoides* | ALND | 4 | 18 | 29 | 34 |
| *Genera* | Abbervatio | Altitude  500m SL | 750m SL | 1000m SL | 1500m SL |
| *Teratorhabditis* | TR | 10 | 15 | 15 | 11 |
| *Mesorhabditis* | MR | 11 | 17 | 18 | 13 |
| *Pelodera* | PD | 8 | 16 | 17 | 18 |
| *Diploscapter* | DS | 12 | 17 | 16 | 15 |
| *Diplogastrid* | DG | 13 | 19 | 22 | 20 |
| *Curviditis* | CD | 9 | 14 | 14 | 10 |
| *Bunonema* | BN | 7 | 13 | 15 | 8 |
| *Rhabditis* | RB | 12 | 14 | 15 | 7 |
| *Protorhabditis* | PR | 10 | 15 | 20 | 14 |
| *Cephalobus* | CP | 7 | 11 | 14 | 10 |
| *Cuticularia* | CC | 10 | 16 | 22 | 18 |
| *Acrobeloides* | ABD | 12 | 18 | 14 | 14 |
| *Acrobelus* | AB | 13 | 20 | 20 | 14 |
| *Rhabditophanes* | RBP | 6 | 14 | 16 | 12 |
| *Bursilla* | BS | 4 | 9 | 16 | 8 |
| *Rhabpanus* | RP | 8 | 12 | 12 | 10 |
| *Cruznema* | CZ | 5 | 9 | 8 | 8 |
| *Eucephalobus* | EPB | 6 | 9 | 8 | 7 |
| *Mononchus* | MNN | 5 | 7 | 9 | 13 |
| *Coomansus* | CMS | 5 | 8 | 10 | 16 |
| *Discolaimus* | DCM | 6 | 8 | 13 | 18 |
| *Clarkus* | CK | 5 | 12 | 15 | 18 |
| *Mesodiplogasteroides* | MDG | 7 | 10 | 17 | 20 |
| *prionchulus* | PNL | 8 | 12 | 16 | 19 |
| *Mylonchulus* | MNL | 8 | 13 | 15 | 18 |
| *Anatonchus* | ANC | 9 | 14 | 14 | 17 |
| *Miconchulus* | MCL | 9 | 10 | 15 | 19 |
| *Longidorus* | LD | 4 | 6 | 14 | 13 |
| *Tylenchus* | TC | 5 | 8 | 17 | 20 |
| *Heterodera* | HD | 2 | 5 | 7 | 7 |
| *Hirschmanniella* | HCM | 4 | 10 | 16 | 10 |
| *Meloidogyne* | MDG | 5 | 13 | 16 | 9 |
| *Paratylenchus* | PTC | 4 | 7 | 10 | 9 |
| *Rotylenchus* | RTC | 6 | 9 | 15 | 10 |
| *Ditylenchus* | DTC | 5 | 8 | 16 | 14 |

| *Helicotylenchus* | HTC | 5 | 8 | 13 | 9 |
| --- | --- | --- | --- | --- | --- |
| *Hexatylenchus* | HXTC | 6 | 8 | 12 | 7 |
| *Globodera* | GBD | 1 | 2 | 3 | 28 |
| *Dorylaimus* | DLM | 8 | 13 | 16 | 17 |
| *Mesodorylaimus* | MDLM | 8 | 14 | 16 | 18 |
| *Eudorylaimus* | EDLM | 7 | 8 | 12 | 24 |
| *Dorylaimellus* | DAL | 8 | 12 | 16 | 20 |
| *Dorylaimoides* | DAD | 11 | 14 | 17 | 21 |
| *Tylencholaimus* | TCM | 9 | 13 | 18 | 22 |
| *Aphelenchus* | ALN | 8 | 13 | 17 | 24 |
| *Aphelenchoides* | ALND | 7 | 9 | 15 | 25 |
| *Genera* |  | Moisture  <25% | <30% | <35% | <40% |
| *Teratorhabditis* | TR | 10 | 15 | 9 | 5 |
| *Mesorhabditis* | MR | 14 | 17 | 13 | 9 |
| *Pelodera* | PD | 11 | 14 | 10 | 4 |
| *Diploscapter* | DS | 16 | 19 | 14 | 10 |
| *Diplogastrid* | DG | 18 | 22 | 15 | 12 |
| *Curviditis* | CD | 12 | 16 | 9 | 7 |
| *Bunonema* | BN | 11 | 13 | 10 | 5 |
| *Rhabditis* | RB | 14 | 23 | 20 | 18 |
| *Protorhabditis* | PR | 16 | 18 | 14 | 10 |
| *Cephalobus* | CP | 12 | 14 | 11 | 8 |
| *Cuticularia* | CC | 19 | 24 | 16 | 13 |
| *Acrobeloides* | ABD | 7 | 10 | 12 | 15 |
| *Acrobelus* | AB | 8 | 13 | 7 | 2 |
| *Rhabditophanes* | RBP | 9 | 15 | 8 | 5 |
| *Bursilla* | BS | 13 | 18 | 10 | 6 |
| *Rhabpanus* | RP | 12 | 17 | 11 | 5 |
| *Cruznema* | CZ | 9 | 13 | 8 | 3 |
| *Eucephalobus* | EPB | 10 | 14 | 7 | 2 |
| *Mononchus* | MNN | 12 | 13 | 15 | 7 |
| *Coomansus* | CMS | 7 | 8 | 11 | 4 |
| *Discolaimus* | DCM | 9 | 10 | 11 | 6 |
| *Clarkus* | CK | 8 | 11 | 11 | 7 |
| *Mesodiplogasteroides* | MDG | 7 | 9 | 12 | 6 |
| *Prionchulus* | PNL | 9 | 11 | 12 | 8 |
| *Mylonchulus* | MNL | 11 | 19 | 15 | 7 |
| *Anatonchus* | ANC | 10 | 12 | 13 | 7 |
| *Miconchulus* | MCL | 11 | 13 | 14 | 6 |
| *Longidorus* | LD | 9 | 13 | 15 | 19 |
| *Tylenchus* | TC | 13 | 14 | 14 | 14 |
| *Heterodera* | HD | 9 | 9 | 8 | 7 |
| *Hirschmanniella* | HCM | 8 | 13 | 16 | 18 |
| *Meloidogyne* | MDG | 6 | 8 | 14 | 14 |
| *Paratylenchus* | PTC | 9 | 8 | 8 | 9 |
| *Rotylenchus* | RTC | 5 | 7 | 10 | 11 |
| *Ditylenchus* | DTC | 10 | 11 | 12 | 12 |
| *Helicotylenchus* | HTC | 13 | 13 | 15 | 16 |
| *Hexatylenchus* | HXTC | 5 | 8 | 8 | 8 |

| *Globodera* | GBD | 4 | 9 | 14 | 19 |
| --- | --- | --- | --- | --- | --- |
| *Dorylaimus* | DLM | 9 | 12 | 16 | 18 |
| *Mesodorylaimus* | MDLM | 13 | 16 | 17 | 19 |
| *Eudorylaimus* | EDLM | 16 | 18 | 19 | 20 |
| *Dorylaimellus* | DAL | 12 | 13 | 9 | 5 |
| *Dorylaimoides* | DAD | 14 | 15 | 10 | 7 |
| *Tylencholaimus* | TCM | 10 | 14 | 13 | 6 |
| *Aphelenchus* | ALN | 10 | 9 | 7 | 3 |
| *Aphelenchoides* | ALND | 11 | 13 | 9 | 5 |
